# Interaction of Soil pH and Mineralogy Controls Soil Organic Matter Persistence through Changes in the Composition and Amount of Microbial Necromass

**DOI:** 10.1021/acs.est.6c08883

**Published:** 2026-07-29

**Authors:** Yuan Liu, Violeta Matus-Acuña, Lisa K. Tiemann

**Affiliations:** † Department of Plant, Soil and Microbial Sciences, 3078 Michigan State University, East Lansing, Michigan 48824, United States; ‡ Physical and Life Sciences Directorate, 4578 Lawrence Livermore National Laboratory, Livermore, California 94450, United States

**Keywords:** mineral-associated organic matter, pH gradient, carbon use efficiency, necromass, functional group, thermal stability

## Abstract

Microbial necromass–mineral associations are key to long-term soil organic matter (SOM) persistence. However, how soil pH and mineralogy interact to regulate SOM stability remains poorly understood. Here, we used artificial soils to test how three clay minerals (bentonite, kaolinite, and goethite), adjusted to four pH levels (5–8), affect microbial activity (respiration), microbial physiology (carbon use efficiency, CUE), microbial-derived residue material (necromass), and the formation and stability of mineral-associated organic matter (MAOM). Artificial soils were inoculated with a rhizosphere-derived microbial community cultured under the same pH conditions and on two representative simulated exudate types (organic acids and carbohydrates) and incubated for 6 weeks. In two complementary experiments, we added necromass from known microbial taxa to the same minerals across pH levels to isolate the role of necromass chemistry and loading. We found that soil pH shaped MAOM chemistry by altering microbial activity and necromass composition. In interaction with mineral type, pH also controlled MAOM thermal stability. Higher necromass loading weakened mineral-organic bonding, reducing MAOM stability, consistent with zonal mineral–organic interaction models. Our results demonstrate that microbial activity, rather than carbon use efficiency, better predicts MAOM formation and that pH-dependent necromass composition and loading govern MAOM persistence. These findings advance mechanistic understanding of SOM stabilization and have implications for predicting soil carbon dynamics under shifting environmental conditions.

## Graphical abstract

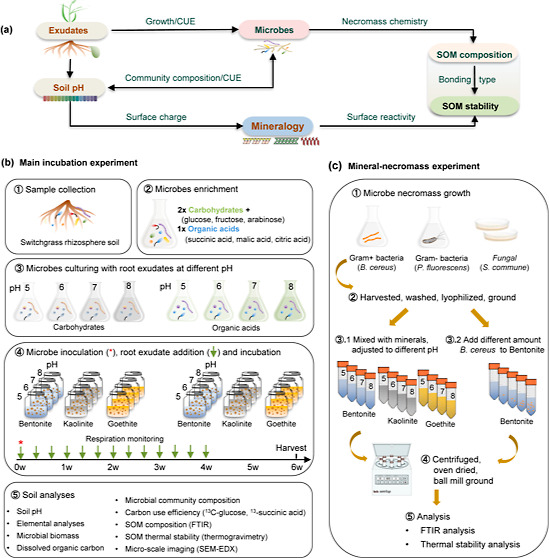

## 1 Introduction

Soil organic matter (SOM), the largest carbon (C) pool in terrestrial ecosystems,^1^ plays a crucial role in nutrient cycling, soil health, and climate mitigation.^2^ Mineral-associated organic matter (MAOM) accounts for over 65% of total SOM.^3,4^ It may determine the rate at which ecosystems respond to climate change.^5^ Long-term MAOM persistence is linked to the sorption of organic matter (OM) onto reactive mineral surfaces,^1,6,7^ which limits microbial decomposition via physical protection or energy constraints.^8–10^ Understanding the mechanisms governing MAOM formation and stability is essential for improving soil management and predicting SOM responses to environmental change.^11,12^ However, how abiotic factors such as soil pH and mineralogy interact with microbes and necromass to regulate MAOM persistence remains unclear.

MAOM stabilization involves ligand exchange, cation bridging, hydrogen bonding, and van der Waals forces.^13,14^ Yet, studies provide inconsistent findings on the role of mineralogy in MAOM stabilization.^15–17^ Some report that clay mineral type influences microbial- and plant-derived OM accumulation,^18^ while others find no clear link between phyllosilicate mineralogy and organic C concentration.^19^ Another study using model soils highlighted that SOM accumulation is driven by distinct microbial communities more than clay mineralogy.^16^ These discrepancies may stem from ignoring covarying factors like soil pH. pH modifies SOM binding mechanisms, shifting from organo-metal complexation to Ca^2+^ cation bridging,^20^ and alters both OM functional groups^14,21,22^ and mineral surface reactivity.^23–25^ Lab batch experiments show extracellular polymeric substances (EPS) sorption decrease with increasing pH, likely due to reduced electrostatic interactions or changes in compound speciation.^24,26^

Microbial necromass is a recognized contributor to SOM formation,^16,27,28^ yet its specific components and stabilization pathways remain underexplored. pH is one of the most important abiotic controls on microbial growth,^29^ microbial community composition and diversity,^30,31^ and carbon use efficiency (CUE).^32,33^ pH change can also affect the amount and/or diversity of cell wall components (functional groups) that will be associated with reactive mineral sites.^16,34,35^ Studies have shown that cell wall chemistry is important, affecting both necromass–necromass interactions as well as necromass retention on mineral surfaces.^35^ These microbial traits can directly impact MAOM composition and thermal stability.^36^

In addition to soil pH, low-molecular-weight root exudates have also been found to play an important role in SOM formation and stability.^34,37^ As important substrates for microbial growth and precursors for SOM formation, root exudates have been found to be more efficient in contributing to SOM formation compared to plant litter.^38^ Differences in root exudate chemistry can affect not only the efficiency with which microbes metabolize exudates but also exudate absorption affinity on mineral surfaces and thus availability to microbes.^32^ Lab and field studies have shown that glucose can have a higher SOM formation efficiency than organic acids,^34,37,39^ while another study found inconsistent results, where organic acid additions led to net MAOM accumulation, while simple sugars (e.g., glucose) increased MAOM turnover (both formation and loss) without altering the size of the MAOM pool.^40^ These divergent findings may reflect differences in soil pH and mineralogy, which covary with exudate identity and microbial processing.^41^

Given its role in microbial physiology and mineral reactivity, pH has been consistently found to be an important control of microbially mediated C cycling at different scales.^42^ For example, meta-analyses of long-term trials consistently found significant negative relationships between SOM and pH on a regional scale,^43,44^ and pH was revealed as the most important control on SOM chemical composition in continental-scale studies.^45,46^ Soil pH was also found to positively correlate with the proportion of microbial necromass in total SOM observed on a global scale.^28^ In addition, a significant positive relationship between soil pH and SOM stability was found across a large regional scale;^47^ however, this relationship may be due to the strong correlation between soil pH and other climate factors. Despite the important role of soil pH in controlling SOM formation and stabilization at different scales through indirect mechanisms described above, the degree to which pH and its interaction with mineralogy directly contribute to SOM formation and stabilization remains unclear because soil pH often covaries with other climate or soil properties that correlate with SOC.^18^ Disentangling these effects is essential to improving mechanistic predictions of SOM persistence.

As many factors controlling soil C cycling covary and given the extreme heterogeneity and complexity of SOM in real environments, it is difficult to disentangle the direct interactions between pH and mineralogy with regards to SOM persistence. Therefore, we used artificial soils as model systems to exclude other covarying factors that may have confounding effects on the results (Figure 1). We selected three minerals (bentonite, kaolinite, and goethite), two simulated exudate types (carbohydrates and organic acids), and four pH levels (5–8). We test the following working hypotheses: (1) realized pH conditions would alter microbial processing of added substrates, as reflected by respiration and CUE where available, with corresponding changes in MAOC retention and composition; (2) mineral type would affect MAOM retention and MAOM composition and thermal stability; (3) MAOC retention would vary across the realized pH gradient, consistent with pH-dependent mineral–organic interactions; (4) mineral type would modulate pH effects, producing mineral and pH interactions in MAOC retention and MAOM stability indices;^23,48^ (5) based on the zonal model of organo-mineral interactions, necromass type and loading would affect the composition and thermal stability of the newly formed MAOM; and (6) simulated exudate type would shift MAOM composition and thermal stability through differences in microbial processing and/or direct mineral–exudate interaction.

**Figure 1:**
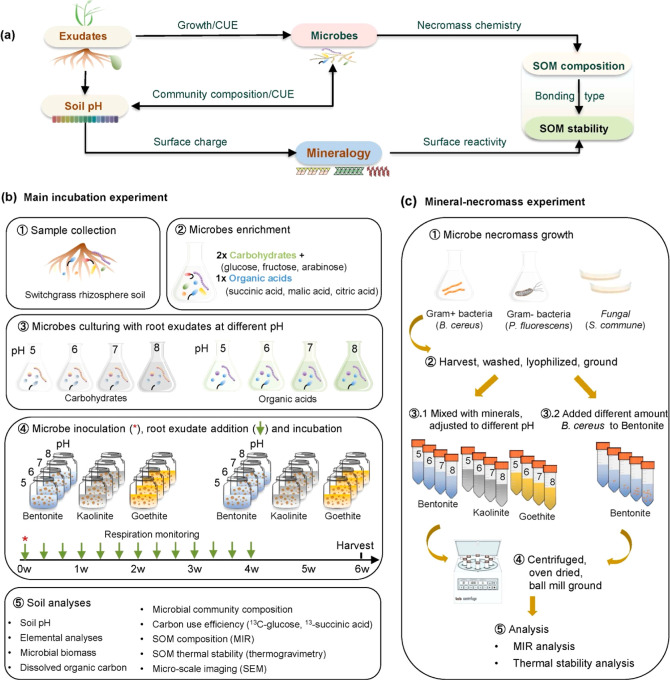
(a) Conceptual framework of the interaction between soil pH, root exudates, and mineralogy on soil organic matter (SOM) composition and stability; (b) experimental setup of incubation with different minerals and root exudates at different pH levels; and (c) two complementary experiments to test the necromass chemistry and necromass loading amount on the composition and thermal stability of mineral-associated organic matter.

## 2 Materials and Methods

### 2.1 Main Experiment

#### 2.1.1 Experimental Design and Sampling

We conducted two complementary laboratory experiments to test how soil pH and mineralogy interact to influence microbial activity, necromass retention, and the stability of mineral-associated organic matter (MAOM). In this manuscript, “microbial necromass” refers broadly to microbially derived cellular residues generated during microbial turnover. Necromass was not quantified using amino-sugar biomarkers; therefore, “necromass” is used here as an operational term. In the main experiment, we constructed artificial soils using quartz sand and one of three minerals (bentonite, kaolinite, or goethite), each adjusted to four pH levels (5–8). Quartz sand consisted of a mixture of particles ranging in size from 0.074 to 1.2 mm (10%: 0.074 mm, 20%: 0.125 mm, 30%: 0.21 mm, 25%: 0.84 mm, : 1.2 mm) to produce a more realistic soil particle size distribution.^200^ Minerals were purchased from Sigma-Aldrich and used as received as fine powders; supplier-reported particle-size metrics (and literature-based size data for goethite) are provided in the Supporting Information (Table S1). All mesocosms contained 100 g dry-weight-equivalent artificial soil (15% mineral + 85% quartz sand); all amendments and response variables were calculated/normalized on a dry-mass basis. MAOM was operationally defined by the <53 μm fraction based on the sieve threshold used for physical fractionation. Soils were inoculated with a rhizosphere-derived microbial community cultured under the same pH and substrate conditions. Each mesocosm received one of two artificial simulated exudate cocktails (carbohydrates or organic acids), with 5 replicate jars per treatment. Simulated exudate cocktails were added as aqueous solutions (2 mg C g^–1^ dry soil); detailed composition and preparation are provided in the SI. This resulted in 120 microcosms (3 minerals × 4 pH levels × 2 exudate types × 5 replicates). Soils were incubated for 6 weeks, including 4 weeks of simulated exudate additions followed by 2 weeks of substrate depletion. After incubation, MAOM was isolated from the mineral fraction and analyzed for organic composition using mid-infrared spectroscopy (MIR) and for thermal stability using thermogravimetric analysis (TGA). Further details, including soil preparation, microbial culturing, pH adjustments, incubation conditions, MAOM composition, and thermal stability characterization, are provided in the Supporting Information.

#### 2.1.2 Soil Respiration, pH, Microbial Biomass, and Microbial Carbon Use Efficiency (CUE)

Soil respiration rates were monitored before and after each simulated exudate cocktail addition (Figure S4) using an infrared gas analyzer (IRGA; LI-820, LI-Cor Biosciences, Lincoln, NE, USA). Headspace gas samples (1 mL) were taken from sealed mesocosms, and respiration rates were calculated by interpolation between time points. Mesocosms were vented/equilibrated to atmospheric pressure to avoid sustained under-pressure and O_2_ limitation prior to each new C-substrate addition. Cumulative CO_2_–C was estimated based on daily rates and time intervals between additions.

Soil pH was measured at the start and end of incubation in a 1:2.5 (w/v; soil/deionized water) suspension using a calibrated pH meter (SevenEasy S20, Mettler Toledo, OH, USA). Artificial soils were adjusted to target pH values before incubation. Because continuous pH monitoring would have required repeated disturbance of the replicated mesocosms, pH was not monitored over time. We therefore interpret pH effects based on the measured start and end point pH values, which represent realized pH conditions during the incubation. Microbial biomass C (MBC) and N (MBN) in the soil were determined using the chloroform-fumigation-extraction method.^49^ Briefly, 40 mL of 0.5 M K_2_SO_4_ was added to 8 g of dry mass equivalent soil, one unfumigated and one directly fumigated for 24 h with 2 mL of ethanol-free chloroform to lyse microbial cells. Total organic C and N in the resulting fumigated and unfumigated soil extracts were then determined by using a Vario Select TOC/TN analyzer (Elementar, Ronkonkoma, NY). The MBC and MBN pools were calculated as the difference in organic C and N between the fumigated and unfumigated samples, divided by a correction factor of 0.45.^50^

Microbial CUE at the end of incubation was determined by adding ^13^C-labeled glucose or succinic acid and measuring ^13^C incorporated into MBC and respired as ^13^CO_2_–C.^51^ Details of methods and calculations are provided in Supporting Information. The thermal stability of the MAOM was investigated using thermogravimetric (TG) analysis using a TGA/DSC 1 STAR system thermogravimetric analyzer (Mettler Toledo, Columbus, Ohio, USA). We calculated the temperature at which half of the mass was lost during TG analysis from 190 to 800 °C (TG-T50) to represent the thermal stability of MAOM from each artificial soil sample.^201^

#### 2.1.3 Microstructural Characterization by SEM

To qualitatively visualize mineral-microbial associations at the microscale, we selected three representative artificial soil samples from the carbohydrate treatment at pH 5, a condition that exhibited clear contrasts among minerals in other analyses, with one sample from each mineral type for scanning electron microscopy (SEM) at the Michigan State University Center for Advanced Microscopy. This targeted selection was intended to provide illustrative, qualitative context rather than quantitative comparisons across the full factorial design. In brief, SEM images were obtained directly from air-dried samples by using a JSM 6610LV scanning electron microscope set on low-vacuum mode, which enables examination of uncoated, nonconducting samples.

### 2.2 Complementary Necromass–Mineral Association Experiment

To further isolate the role of microbial necromass chemistry and loading on MAOM formation and stability, we conducted two simplified laboratory experiments. In the first, we selected representative Gram-positive bacteria, Gram-negative bacteria, and fungi to generate chemically distinct necromass endmembers that reflect major microbial groups in soils. These groups differ in cell-envelope polymers relevant to mineral association (e.g., peptidoglycan/teichoic acids in Gram-positive bacteria; lipopolysaccharides in Gram-negative bacteria; and chitin- and melanin-associated polymers in fungi), providing ecologically relevant contrasts for evaluating necromass–mineral interactions. Necromass was produced from pure cultures and incubated with clay minerals (bentonite, kaolinite, or goethite) adjusted to four pH levels (5–8). In the second experiment, we systematically varied the loading of bacterial necromass (*Bacillus cereus*) onto bentonite to test how necromass quantity influences MAOM composition and thermal stability. In both experiments, samples were analyzed using MIR for necromass chemistry and thermogravimetric analysis (TGA) for thermal stability. Full experimental details, including microbial strain culturing, mineral preparation, pH adjustments, mixing protocols, and analytical procedures, are provided in the Supporting Information.

### 2.3 Statistical Analysis

All statistical analyses were performed using the software R version 4.1.2 (www.r-project.org). Data were tested for normality (Shapiro–Wilk test) and homogeneity of variance (Levene’s test), and variables were transformed where necessary to meet model assumptions of a normal distribution.^41^ Three-way analysis of variance (ANOVA) using linear mixed-effect models in the R package *nlme* was used to test the effect of mineralogy, pH, simulated exudate type, and their interactions for the variables: pH, respiration, MBC, DOC, CUE, MAOC, POM, thermal stability, and MAOM compound classes, including aliphatic, carboxyl, aromatic, and the ratio of aromatic to aliphatic, and when the overall model indicated a significant effect (*p* < 0.05), pairwise comparisons were evaluated using Tukey’s HSD *post hoc* tests. Models were fitted with mineralogy, pH, and exudate type as fixed effects (including interactions), and jar replicate was included as a random intercept to account for replication. We used a similar approach to investigate fixed effects of and interactions between necromass type, mineralogy, and pH on the composition and thermal stability of mineral-associated necromass. Pearson’s correlations between pH, microbial function indicators (respiration and CUE, where available), organic matter compounds (aliphatic, carboxyl, aromatic, and the ratio of aromatic to aliphatic), and thermal stability were examined with the *corrplot* package in R. Interactions of pH, mineralogy, and simulated exudate type on SOM composition from the main experiment, as well as differences in the chemical composition of the three necromass types in the complementary experiment, were evaluated from MIR spectra using principal coordinates analysis (PCoA), with treatment effects tested by PERMANOVA (Bray–Curtis dissimilarity; 9999 permutations) using the adonis2 function in the *vegan* R package. Path analysis using the *lavaan* package in R was used to evaluate the conceptual pathways linking soil pH, simulated exudates, and mineralogy to MAOC and thermal stability via microbial processing indicators and MAOM composition. Amino sugars were not measured, and CUE was quantifiable only for carbohydrate treatments; therefore, the primary path model was fit using variables available across the full factorial data set, and a carbohydrate-only sensitivity model including CUE was evaluated in Figure S2. Mineralogy was treated as a categorical fixed factor with three levels (bentonite, kaolinite, goethite) in path analysis. Model fit was evaluated using multiple indices (χ^2^, RMSEA, SRMR, and comparative fit indices), and standardized path coefficients were reported.

## 3 Results

### 3.1 Microbial Community Function (Respiration, Biomass, CUE) among Different Treatments

After incubation, artificial soil pH decreased significantly compared with its original level (Table 1 and Figure S3c), with the greatest decrease at a high (pH 8) pH level. However, even with these uneven decreases, the pH gradient across treatments was maintained, except for the bentonite mineral with carbohydrate treatment that had a lower final pH when starting at pH 7 when compared to starting at pH 6. This could be due to the observed higher fungal growth (visible hyphae and mycelium) in the bentonite carbohydrate treatment at pH 7 (data not shown).

**Table 1:** Summary (F-Values) of Three-Way ANOVA for the Effects of Mineral, Root Exudates (REs), pH Treatment, and Their Interactions on Soil Properties and Soil Organic Matter Composition and Thermal Stability. *, **, and *** Represent Significance at *p* < 0.05, *p* < 0.01, and *p* < 0.001, Respectively

variables	mineral	root exudates (REs)	pH	mineral * RE	mineral * pH	RE * pH	mineral * RE * pH
pH_start_	0.82	0.006	2843.62***	5.34**	7.30***	0.16	1.46
pH_end_	39.18***	154.37***	1807.87***	95.89***	2.67*	175.06***	12.87***
respiration	120.15***	8.46**	107.69***	16.55***	21.08***	3.40*	10.50***
MBC	6.61**	/	0.45	/	0.057	/	/
CUE	86.65***	/	7.68***	/	3.82**	/	/
DOC	74.75***	132.19***	6.51***	32.15***	2.56*	1.97	0.7
MAOC	372.59***	122.31***	65.5***	22.36***	117.31***	5.60**	14.24***
TGA-T_50_	5313.9***	95.27***	19.29***	107.62***	14.84***	2.22	3.20
aliphatic	176.03***	85.75***	13.80***	35.71***	4.92***	0.89	1.05
carboxyl	174.96***	2.00	1.26	14.18***	0.70	0.60	1.91
aromatic	150.93***	0.21	0.71	1.14	0.26	0.31	2.30
POM	105.91***	0.34	18.44***	0.089	11.43***	20.39***	14.59***

There were significant effects of mineralogy, pH, simulated exudate type, and their interactions on microbial respiration rates averaged across the 6 week incubation period (Table 1; Figures 2a and S4). Within the organic acid (OA) simulated exudates, bentonite and kaolinite had the greatest respiration rates at pH 5 compared to pH 7 and 8. Within the carbohydrate-simulated exudate treatment, bentonite had the greatest respiration at pH 5 compared to pH 6, 7, and 8, while kaolinite had greater respiration rates at pH 5 and 7 compared to pH 8. Within both the OA- and carbohydrate-simulated exudate treatments, respiration rates were also influenced by the mineral type. We found greater respiration rates in bentonite at OA pH 6 and in carbohydrate at pH 5 and 6 compared to kaolinite or goethite OA at the same pH levels in OA and carbohydrate treatments. With data only from the carbohydrate treatment, MBC was significantly affected only by mineral type but not pH treatment. Bentonite and goethite had greater MBC compared to kaolinite, while there were no significant differences between bentonite and goethite (Figure 2b). DOC was significantly impacted by mineralogy, pH, simulated exudate type, and interactions (Table 1; Figure 2c). In general, pH had less impact on DOC accumulation than mineralogy; bentonite and kaolinite had lower DOC at pH 5 compared to pH 7 and 8 within the OA-simulated exudates; bentonite and goethite at pH 5 have lower DOC within the carbohydrate-simulated exudate treatment. Bentonite had significantly lower DOC than kaolinite and goethite minerals from carbohydrate treatments at all pH levels, and there was significantly less DOC accumulated in bentonite under carbohydrates than in those under OA-simulated exudate treatment at all pH levels (Figure 2c). With data only from the carbohydrate-simulated exudate treatment, we found that microbial CUE was significantly affected by mineralogy, pH, and their interactions (Table 1; Figure 2d). Bentonite had significantly lower CUE at pH 5 and 7 compared to pH 6 and 8; goethite had lower CUE at pH 5 and 6 compared to pH 7 and 8, but the differences were not significant. Bentonite had a significantly lower CUE than kaolinite and goethite at pH 5 and 7. Microbial CUE tends to increase with increasing pH in all mineral types (Figure S5), where this trend was only significant in bentonite (*p* < 0.05) and marginally significant in kaolinite (*p* = 0.07) and goethite treatments (*p* = 0.08).

**Figure 2:**
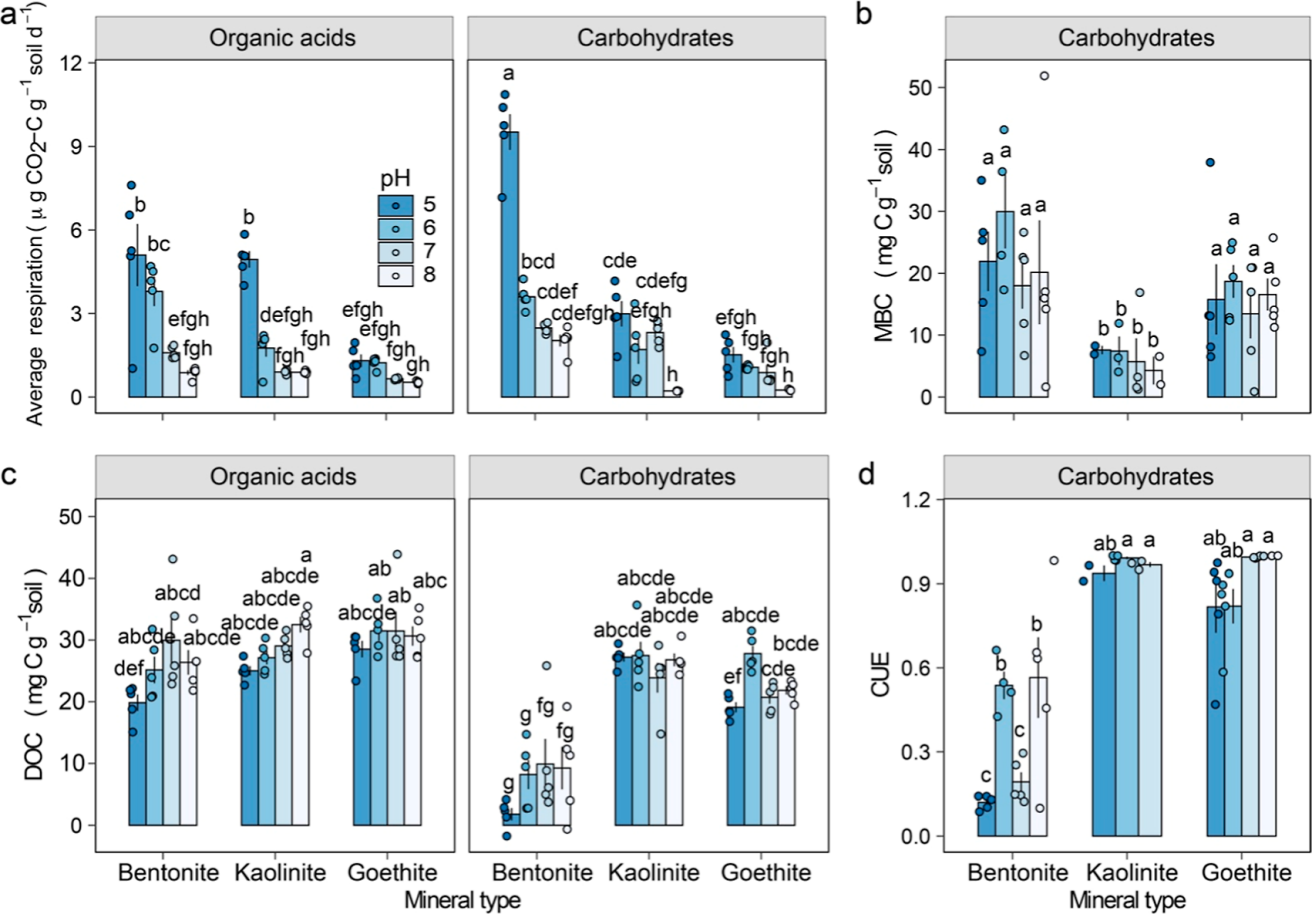
Average microbial respiration rate over the 6 week incubation period (a); dissolved organic carbon (DOC) (b); microbial biomass carbon (MBC) (c); and microbial carbon use efficiency (CUE) (d) in different minerals at different pH levels. Lowercase letters indicate significant differences between different minerals, pH levels, and root exudate types. Data are mean values ± SE (*n* = 5).

### 3.2 Chemistry Composition, C Content, and Thermal Stability of MAOM

PCoA indicated that MAOM chemistry composition characterized with MIR differed significantly among mineral type, pH, and simulated exudate treatments (Figure 3a–c). More specifically, we focused on three spectral peaks that reflect aliphatic, carboxyl, and aromatic compounds of MAOM (Figure S6). There were significant mineralogy, pH, and simulated exudate types on aliphatic compounds (Table 1). Within the OA-simulated exudates, the aliphatic abundance of bentonite and kaolinite minerals decreased with increasing pH (Figure 3d). Within the carbohydrate-simulated exudates, aliphatic abundance in bentonite at pH 5 was greater compared to pH 6, 7, and 8. Within both simulated exudate treatments, aliphatic accumulation was also influenced by the mineral type. We found greater aliphatic accumulation in goethite at pH 6, 7, and 8 compared to bentonite and kaolinite at the same pH levels in OA treatments, while kaolinite had significantly lower aliphatic abundances than bentonite and goethite at all pH levels. Carboxyl and aromatic accumulation followed the same trend and was only significantly influenced by mineral type but not pH and simulated exudate types (Table 1; Figure 3d). Goethite had higher carboxyl and aromatic compound accumulation than bentonite and kaolinite at both simulated exudate treatments.

**Figure 3:**
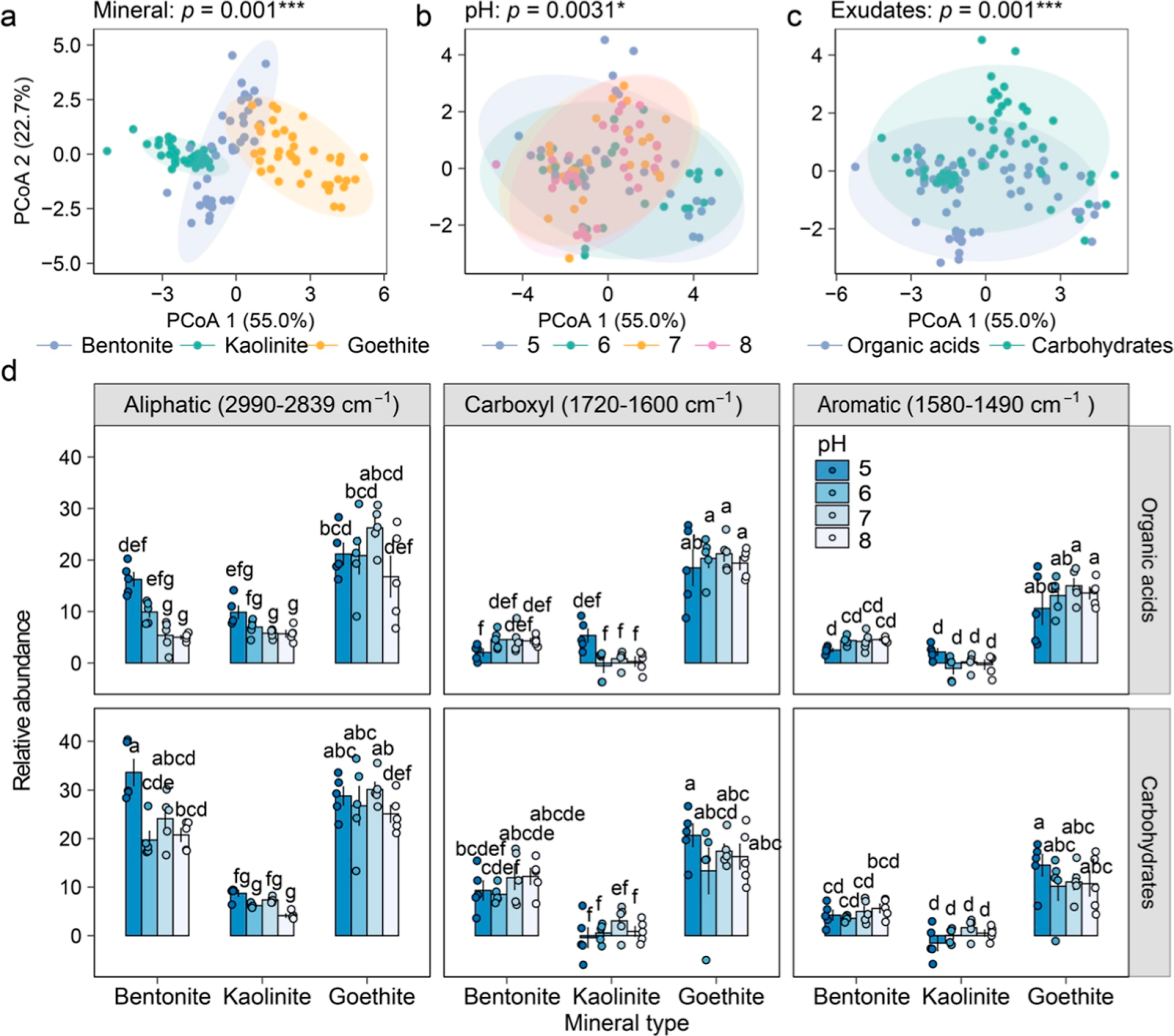
Principal coordinates analysis (PCoA) of mineral-associated organic matter (MAOM) composition characterized by mid-infrared spectroscopy (MIR), grouped by (a) mineral type, (b) pH treatment, and (c) simulated exudate type. (d) Relative abundance of selected MIR functional group indices in MAOM across minerals × pH × exudate treatments. Data are the mean ± SE (*n* = 5). Different lowercase letters indicate statistically significant differences among groups within each panel (*p* < 0.05; Tukey posthoc tests following linear mixed-effect models).

MAOC accumulation was significantly affected by mineralogy, pH, simulated exudates, and their interactions (Table 1 and Figure 4a). Bentonite and kaolinite at pH 5 had significantly higher MAOC accumulation compared to pH 7 and 8 within the OA-simulated exudate treatment, while only bentonite at pH 5 had significantly higher MAOC accumulation than the other pH levels. Within both the OA- and carbohydrate-simulated exudate treatments, MAOC accumulation was also influenced by the mineral type. Bentonite at pH 5 and 6 as well as kaolinite at pH 5 had significantly higher MAOC accumulation than goethite at the same pH level within OA-simulated exudate treatments, while only bentonite accumulated significantly higher MAOC than both kaolinite and goethite at all pH levels within carbohydrate-simulated exudate treatments. In addition, bentonite accumulated significantly higher MAOC under carbohydrate treatment than under OA treatment at pH 5.

**Figure 4:**
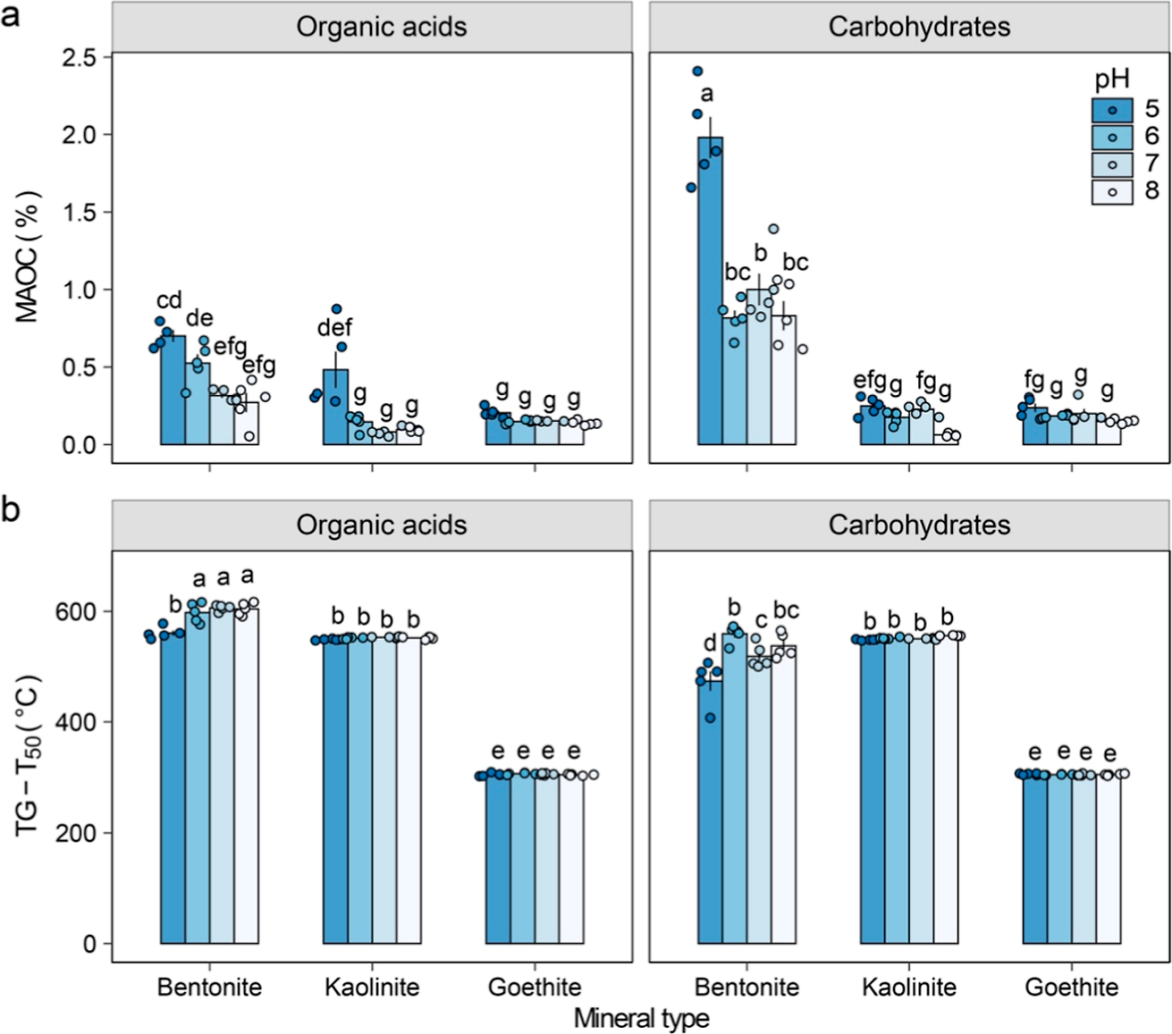
Percentage of mineral-associated organic carbon (MAOC) (a) accumulated during incubation and its stability estimated by the thermal stability indices (TG-T_50_) (b) for three minerals at different pH levels and root exudate treatments. Lowercase letters indicate significant differences among different minerals, pH levels, and root exudate types. Data are mean values ± SE (*n* = 5).

Once associated with minerals, the thermal stability indices of MAOM (TG-T_50_) were significantly affected by mineralogy, pH, and simulated exudate types (Table 1; Figure 4b). Bentonite at pH 5 had significantly lower TG-T_50_ than pH 6, 7, and 8 at both simulated exudate treatments. Bentonite and kaolinite both had significantly higher TG-T_50_ than goethite at all pH levels under both simulated exudate treatments. In addition, only the TG-T_50_ in bentonite under carbohydrate was significantly lower than that under the OA treatment at all pH levels.

### 3.3 Relations between Microbial Community Activity and the Accumulation, Composition, and Thermal Stability of MAOC

There were significant correlations between soil pH, microbial activity (indicated by microbial respiration, *R*_s_), CUE, the content, composition, and the thermal stability of MAOM (Figure 5), and the strength of those correlations depends on soil mineralogy (Figure S7a–d). Across all mineral types, MAOC were positively correlated with microbial activity and aliphatic compounds but negatively correlated with pH_end_ and CUE (Figures S6 and S7). Most correlations with MAOC and TG-T_50_ under goethite treatment were not significant. For bentonite and kaolinite, MAOC was positively correlated with aliphatic and aromatic compounds but negatively correlated with TG-T_50,_ pH, and CUE (Figure S7b,c). In contrast, TG-T_50_ was negatively correlated with aliphatic compounds and MAOC but positively correlated with pH and CUE. TG-T_50_ increased significantly with increasing pH but decreased significantly with increasing aliphatic compounds for both simulated exudate types for both bentonite and kaolinite minerals, but goethite mineral (Figure S9). Only in bentonite mineral, TG-T_50_ increased significantly with increasing ratio of carboxyl and aliphatic compounds, as well as the ratio of aromatic and aliphatic compounds under both exudates’ treatments (Figure S10).

**Figure 5:**
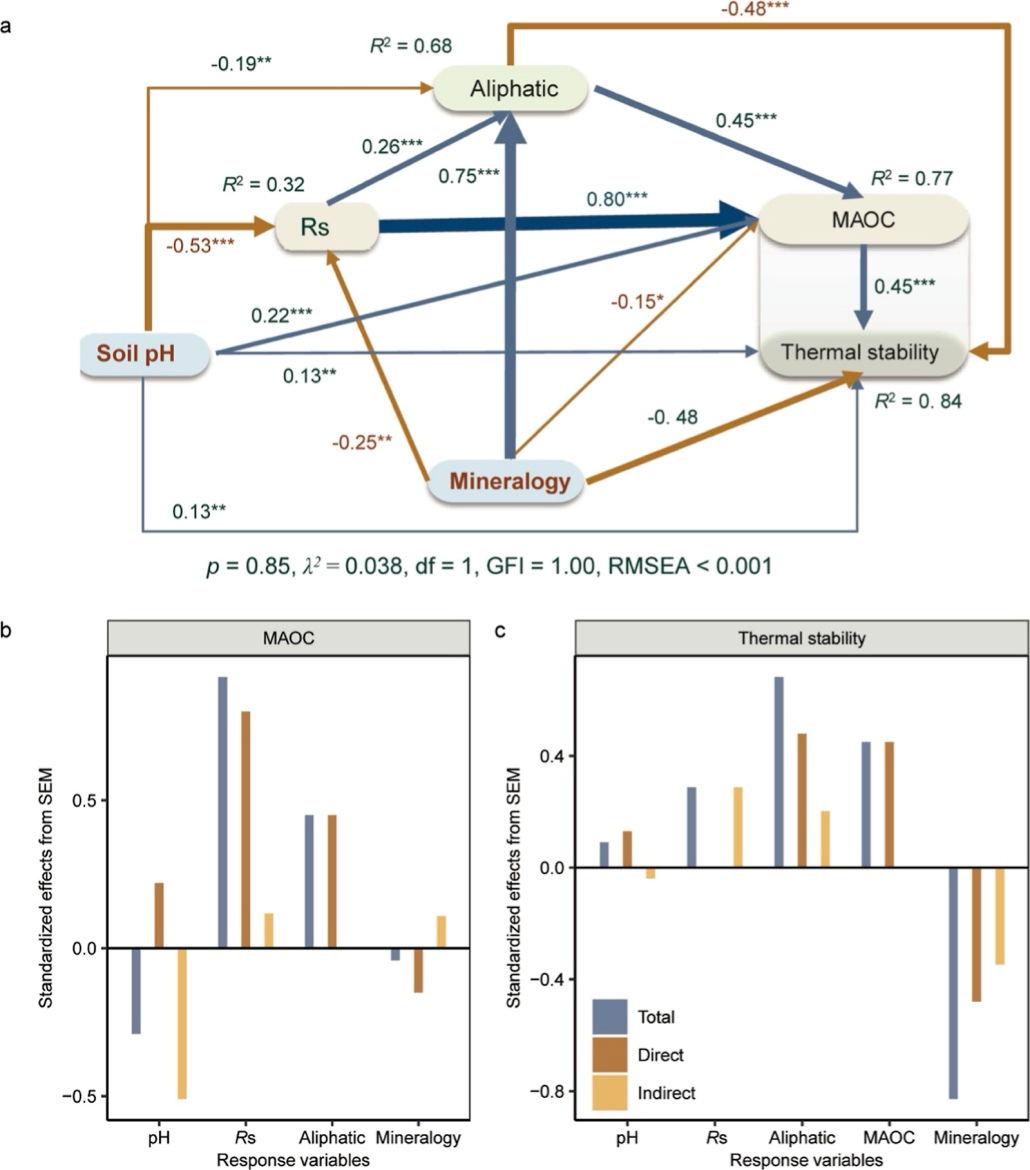
Path analysis testing the controls that regulate the content and thermal stability of mineral-associated organic matter (a), standardized direct and indirect effects from the path analysis of different factors on mineral-associated organic carbon (MAOC) (b), and thermal stability (c), respectively. *, **, and *** indicate significant differences at the *p* < 0.1, *p* < 0.05, and *p* < 0.01 levels, respectively. *R*_s_: average microbial respiration rate during the incubation as an indicator of microbial activity.

Path analysis indicated that pH, mineralogy, and their interactions affected the accumulation and thermal stability of MAOM through their direct influences on microbial activity (indicated by respiration rate) and necromass chemistry (aliphatic compounds). Overall, the combination of abiotic factors (pH, mineralogy) and biotic factors (microbial activity) together explained 68% of the variation in aliphatic compound content and 77% of the variation in MAOC. Those abiotic and biotic factors combined with aliphatic compounds together explained 84% of the variation in the thermal stability of MAOM (Figure 5a). For MAOC, the microbial activity contributes to the most variation of MAOC through its direct effect (Figure 5b), while the mineralogy contributes the most variation of thermal stability of MAOM (Figure 5c).

### 3.4 Necromass Type and Its Interaction with pH and Mineralogy on MAOM Composition and Thermal Stability

Microbial necromass chemistry differed significantly among different microbial groups (Figure S11). Fungal necromass had the lowest relative abundance of aliphatic, carboxyl, and aromatic compounds compared with Gram-positive and Gram-negative bacterial necromass, whereas the differences were not significant within the two bacterial types. When the microbial necromass was associated with minerals at different pH levels, we found significant interactions among necromass type, mineralogy, and pH on MAOM chemistry composition and their thermal stability (Table S2; Figure 6). Goethite had the highest and kaolinite had the lowest aliphatic accumulation at all pH levels with all necromass types. In contrast, goethite had the lowest carboxyl and aromatic compound accumulation when associated with fungal necromass. pH had a less important role in the retention of necromass on the mineral surface, and only aliphatic and carboxyl compound accumulation in kaolinite associated with bacterial necromass decreased with increasing pH (Figure 6). When averaged across all pH levels, necromass type significantly affected the retention of carboxyl and aromatic compounds but not aliphatic compounds on mineral surfaces (Figure S12). There were significantly fewer carboxyl and aromatic compounds retained on all three minerals when associated with fungal necromass compared with bacterial necromass, whereas the retention of those compounds was not significant within the two bacterial types (Figure S12).

**Figure 6:**
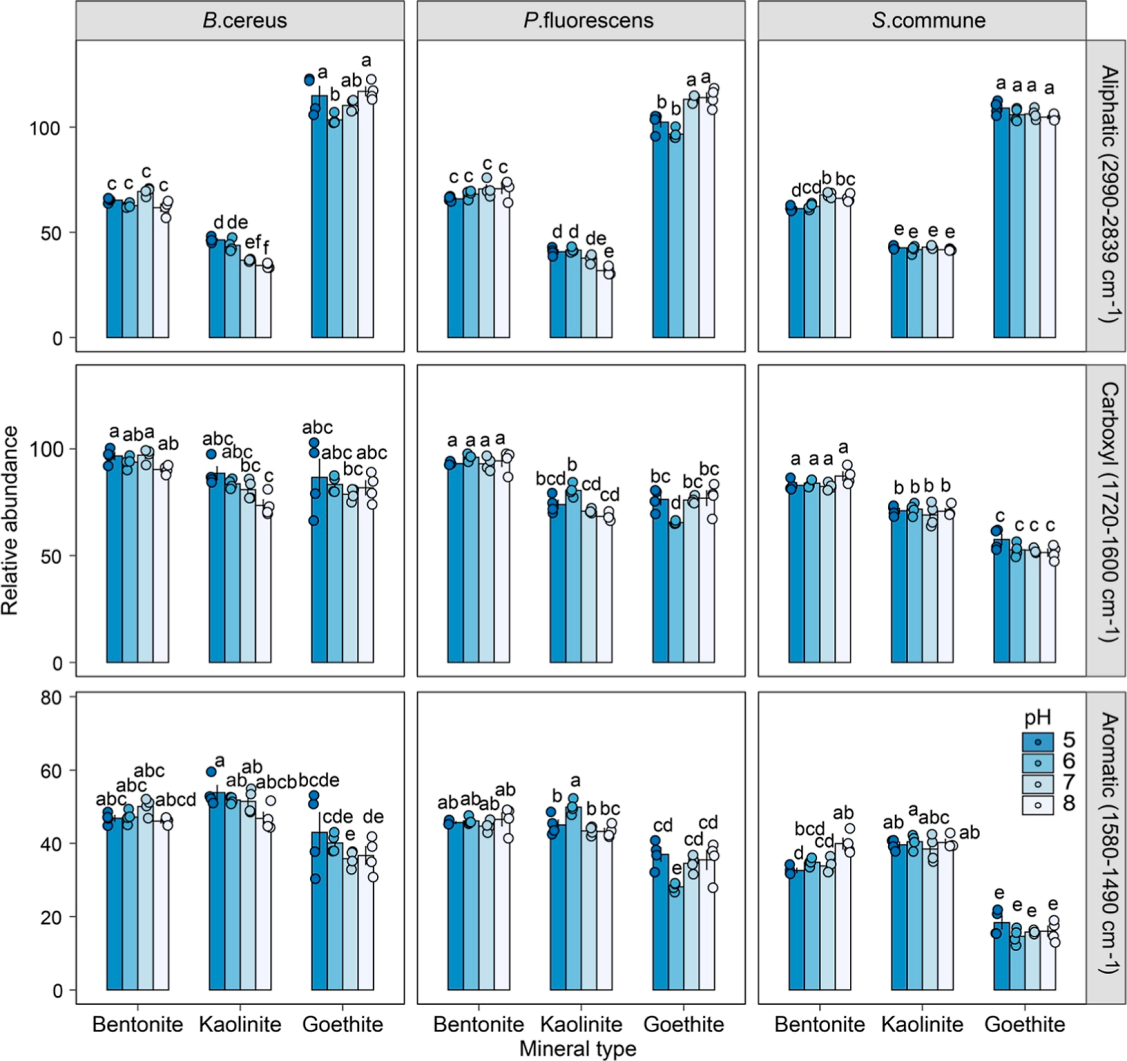
Relative abundance of the functional group of three pure microbial necromasses associated with different minerals at different pH levels examined by MIR. Lowercase letters indicate significant differences between minerals and pH levels within each necromass type. Data are mean values ± SE (*n* = 3).

The interactions between mineralogy and the necromass type significantly affect the thermal stability of the mineral-associated necromass (Table S2). All necromasses had the highest thermal stability once they were associated with kaolinite and the lowest thermal stability associated with goethite (Figure S13). pH had no significant effect on thermal stability once the necromass was associated with minerals (Figure S13). Overall, fungal necromass had lower thermal stability once associated with bentonite and kaolinite minerals than bacterial necromass, but the differences were only significant in bentonite minerals (Figure S13a). There were no significant differences in the thermal stability between two bacterial types. The thermal stability of mineral-associated necromass had significant negative correlations with the relative abundance of aliphatic compounds but positively correlated with the ratio of aromatic to aliphatic (Figure S14). Thermal stability increased significantly with increasing carboxyl and aromatic abundance in both bentonite and kaolinite minerals but not in goethite minerals (Figure S14b,c).

### 3.5 Necromass Loading on the MAOM Composition and Its Thermal Stability

The amounts of bacteria necromass (*B. cereus*) associated with bentonite mineral saturated with increasing necromass loading (Figure S15) and the relative abundance of carboxyl compounds were significantly higher than aliphatic compounds and aromatic compounds (Figure S15b). The necromass loading on minerals significantly affects its thermal stability, with thermal stability decreasing with the loading amount of necromass (Figure S16).

## 4 Discussion

The role of microbial residues in SOM formation has been increasingly recognized,^16,27^ particularly due to their close association with minerals, which promotes their long-term persistence compared to plant-derived compounds.^13^ As such, understanding the factors that regulate microbial physiology is essential for improving our knowledge of SOM stabilization.^52^ Among abiotic factors, pH is known to influence microbial community structure,^30^ growth rates,^29^ and carbon use efficacy (CUE).^33^ Because microbial necromass formation depends on these traits, abiotic regulation of microbial communities likely governs necromass retention^22^ and MAOM formation efficiency.^18^ In addition, pH directly influences the sorption of organic ligands onto minerals.^14^ Although pH is recognized as a driver of soil C cycling at regional^46^ and global scales,^28^ its direct interaction with mineralogy in regulating SOM stability remains insufficiently understood. Using artificial soil as a controlled model system, our experiments isolated the independent and interactive effects of the soil pH and mineralogy on SOM formation and stabilization. We found that pH primarily shaped the chemical composition of newly formed OM through presumed changes in microbial community structure^30,31^ and changes in microbial activity (i.e., respiration) and community physiology (i.e., CUE). These changes, in combination with mineral interactions, govern the thermal stability of MAOM. Unlike some previous studies that emphasized the role of C input chemistry,^16^ our results underscore the stronger influence of mineralogy and its interactions with pH on SOM stabilization and somewhat downplay the chemical composition of C inputs such as simulated exudates. While earlier regional studies showed that soil pH and mineralogy are correlated with SOM stability, these relationships may be confounded by covarying factors such as climate.^47^ Our lab-based artificial-soil system helps separate pH and mineralogical effects from these field covariates. Because pH was measured at the start and end of incubation but not continuously, we interpret treatment effects as responses to realized pH conditions rather than invariant pH values maintained throughout the incubation. Start- and end-point measurements showed that the intended pH gradient was maintained, although some drift occurred (Figure S3).

### 4.1 Effect of pH and Mineralogy on Microbial Physiology

Our results support ***hypothesis 1*** that realized pH conditions affect microbial processing, as reflected by respiration and CUE. These shifts in microbial processing may influence the amount and composition of microbially derived residues contributing to MAOM, although necromass production was not quantified directly. Microbial activity, as measured by average respiration, generally decreased with increasing pH, independent of mineralogy or simulated exudate type (Figure 2). This suggests that a shift in microbial community structure at higher pH levels may reduce activity, consistent with earlier findings.^53^ Although some measured CUE values were relatively high, they remain within the reported range (0.05–0.95).^32,33,54^ We speculate that higher CUE values may arise if microbial-derived compounds are more rapidly bound on mineral surfaces, which could reduce the availability of released dissolved substrates for repeated microbial uptake (“recycling”) and thereby shift carbon partitioning toward biomass relative to respiration.^55^ Mineral type also impacted CUE, with kaolinite and goethite supporting higher CUE than bentonite. Interestingly, only bentonite showed significant pH-related variation in CUE (Figures 2d and S5). Bentonite also supported more fungal growth (data not shown) and accumulated more particulate organic matter (Figure S17). We speculate that stronger adsorption in bentonite reduces substrate availability, increasing microbial energy demands and respiration (Figure 2a) while decreasing CUE (Figure 2d). Despite its lower CUE, bentonite soils accumulated the most MAOC, likely due to greater microbial biomass and activity (Figure 2) and enhanced water retention that^56^ promoted microbial necromass production and preservation.

### 4.2 Effects of pH and Mineralogy on the Formation, Composition, and Stabilization of MAOM

As hypothesized, both pH and mineralogy directly influenced the composition and stability of MAOM, but these effects varied across simulated exudate treatments. MAOC accumulation was primarily driven by interactive effects between mineral type, pH, and simulated exudates such that there were more pronounced mineral and pH effects with carbohydrate-simulated exudates. Surprisingly, goethite accumulated the least MAOC compared with bentonite despite supporting higher CUE (Figure 4), contrary to our ***second hypothesis*** based on its high specific surface area.^16,57,58^ In this study, higher microbial CUE was associated with lower MAOC accumulation, which is also in contrast to previous studies that found positive correlations between CUE and SOC accumulation.^16,52,59^ For example, in an artificial soil system, Kallenbach et al. (2015) found that minerals with a higher CUE correlated with a greater fungal abundance and a higher MAOC content.^16^ However, other studies have also found negative correlations between CUE and MAOC content, like our study. For example, in a ^13^C-labeled greenhouse experiment, CUE was found to be a negative predictor of ^13^C-MAOC in both the detritusphere and the rhizosphere.^60^ A field plant litter decaying experiment also observed a negative correlation between microbial growth efficiency and MAOC content.^61^ Instead of CUE, we found microbial respiration more closely tied to MAOC content with a significant positive correlation between microbial activity (indicated by average respiration rate, Figure S7) and MAOC content, independent of mineral types. Therefore, despite the low CUE and higher respiration, minerals at low pH conditions accumulated the highest MAOC (Figure 4), which supports our ***third hypothesis***. We propose that under low-pH conditions, microbes may inefficiently convert C into biomass, leading to higher respiration (lower CUE) and more byproduct excretion, such as extracellular polymeric substances (EPS), which readily interact with mineral surfaces that contribute to MAOC formation. For example, a recent study found that the higher EPS production in the rhizosphere was positively associated with ^13^C-MAOC.^60^ Goethite-supported microbial activity was low throughout incubation (Figures 2a and S4), possibly due to its antibacterial properties as previously shown since the iron content is much higher than normally found in the real soil; goethite can induce severe membrane damage that inhibits microbial growth.^62^ Therefore, while the CUE was high, respiration and necromass production were low. Overall, our findings suggest that microbial respiration is a better predictor of MAOC accumulation than CUE.

MAOC accumulation in bentonite and kaolinite was strongly correlated with aliphatic compound abundance, while goethite showed no such relationship (Figure S6b–d). Under carbohydrate treatments, bentonite accumulated significantly more MAOC than did other minerals. This pattern coincided with higher microbial activity and biomass and lower measured DOC concentration (Figure 2a,b). Although we cannot distinguish uptake from sorption with these data, lower DOC is consistent with greater microbial uptake/processing of dissolved substrates and/or stronger mineral association of DOC, either of which could contribute to the higher retention of microbially processed C in the operational MAOM fraction. With organic acid-simulated exudates, pH effects were more pronounced in bentonite and kaolinite, where MAOC increased under lower pH (Figure 4a). This aligns with our ***fourth hypothesis*** regarding pH sensitivity of mineral-organic interactions. However, we note that variable-charge minerals such as goethite are often pH-sensitive,^63^ and our results indicate only that goethite showed a weaker net pH response in MAOC under the conditions tested. In contrast, although phyllosilicates have largely permanent basal plane charge, their net interactions with organic acids and microbially processed products may still vary with pH (e.g., due to pH-dependent edge-site chemistry and solution conditions), which could contribute to the stronger pH dependence observed for bentonite and kaolinite in this system. In addition, because exchangeable cation composition was not quantified for the commercial clays, we cannot directly evaluate Ca-bridging capacity; future work measuring CEC and exchangeable cations would help link mineral chemistry to the observed MAOM patterns.

We found that MAOM chemical composition varied with pH, mineral type, and simulated exudates, but their interactions affected individual compound classes differently. In phyllosilicates (bentonite and kaolinite), aliphatic compound abundance decreased with increasing pH (Figure 3d), mirroring MAOC trends (Figure 4a) and likely reflecting reduced necromass sorption. These findings align with studies reporting lower bacterial necromass adsorption at high pH.^25,64^ Carboxyl and aromatic compounds, however, were more strongly influenced by mineral exudate interactions. For example, organic acid promoted carboxyl accumulation in goethite, likely due to the strong ligand exchange between the carboxyl group and the goethite surface compared with the weaker hydrogen bonding with carbohydrate.^65^

The thermal stability of MAOM was primarily determined by mineralogy, with goethite supporting the least stable MAOM (Figure 4b). While bentonite showed some pH- and exudate-related variations, these effects were secondary. MAOM stability increased with pH in bentonite, likely because of a shift in necromass chemistry. More oxidized compounds, particularly carboxyl groups, are more stable and bind more strongly to the mineral surface.^66^ In our results, carboxyl groups associated with bentonite increased (although not significantly) with pH for both simulated exudate treatments (Figure 3d), which might explain the increasing stability with pH (Figure 4b). As SOM undergoes oxidation through microbial activity, the proportion of carboxyl groups increases. Therefore, a higher ratio of carboxyl and aliphatic groups can indicate a higher oxidation status of OM.^67^ The strong correlations between the ratio of carboxyl and aliphatic and the thermal stability of bentonite (Figure S10) suggest the stability of the newly formed MAOC might be related to its oxidation status that shifted with pH.

Goethite-associated MAOM was less thermally stable, likely due to higher relative abundance of aliphatic compounds and lower carboxyl and aromatic content (Figure S9). The aliphatic C–H bonds (alkyl CH_2_^–^ and CH_3_^–^) generally contain high energy^68^ and lower binding strength compared with the carboxyl group.^22^ Therefore, the higher aliphatic relative abundance associated with goethite might explain its lower thermal stability of MAOM (Figure 4). Additionally, goethite’s lower structure stability and transformation to hematite upon heating may further explain its low MAOM thermal stability.^69^

### 4.3 Connections between MAOM Stability, Necromass Loading, and MAOM Chemical Composition

Our second complementary experiment revealed that MAOM thermal stability declines with increasing necromass loading on minerals (Figures S15 and S16). These results are consistent with the zonal organo-mineral interactions,^70^ in which strong organo-mineral bonds dominate at low C loadings, but weaker organic–organic interactions prevail as loading increases.^71^ We found a significant negative correlation between thermal stability and aliphatic compounds in phyllosilicates, but not in goethite minerals (Figure S8). At the same time, only for bentonite and kaolinite, a significant positive correlation between aliphatic compounds and MAOC suggests that aliphatic compounds might indicate the necromass accumulation (Figure S7). Therefore, the negative correlations between thermal stability and aliphatic compounds likely indicate that the thermal stability of MAOM decreases with increasing necromass adsorbed on minerals (Figure S9). This could also partly explain the increase in thermal stability with increasing pH (Figures 4 and S9) because of reduced necromass loading/adsorption (aliphatic compounds, Figure 3) with increasing pH. These relationships suggest that thermal stability declines as necromass accumulation increases.

Shifts in the MAOM stability with pH, particularly for bentonite, may reflect microbial community transitions. We could not directly analyze the microbial community composition in our artificial soil due to the difficulty in extracting DNA. However, many previous studies have shown that pH affects the microbial community composition, with fungi dominating under acidic environments and bacteria generally dominating under alkaline environments.^30^ At the end of incubation, we observed greater hyphal growth and measured greater POM (Figure S17, which mainly consisted of fungal hyphae) in bentonite under acidic conditions (Figure S17). Scanning electron microscopy (SEM) images qualitatively indicate filamentous structures consistent with fungal hyphae on mineral surfaces under lower pH conditions (Figure S18). Because fungi and bacteria can produce residues with distinct chemical characteristics, these observations are consistent with possible pH-associated differences in microbial processing and residue characteristics that may contribute to MAOM composition and stability.^72^ However, SEM is qualitative, was not conducted across the full factorial design, and does not distinguish living biomass from necromass.

A previous study suggested that microbial community composition rather than diversity drives SOM composition and thermal stability.^36^ We tested this idea using pure cultures to assess how different microbial species, producing necromass of varying chemical compositions, would affect the thermal stability of newly formed MAOM (Figure 6). Fungal necromass had a lower abundance of all identified functional groups—aliphatic, aromatic, and carboxyl (Figure S11)—and resulted in less stable MAOM than bacterial necromass in both phyllosilicate minerals (Figure S13). These differences likely stem from lower carboxyl group abundance in fungal residues, which bind less strongly to minerals.^22^ Another study indicates that the stabilization and persistence of organic matter increased with an increasing number of carboxyl functional groups,^66^ suggesting that persistent organic matter is more oxidized.^68^ Therefore, the lower thermal stability of MAOM at lower pH for bentonite in our study could be partly explained by the dominance of fungal necromass with lower carboxyl abundance, and a shift in microbial composition toward the bacteria-dominated community (higher carboxyl abundance) with increasing pH can result in higher thermal stability. Overall, those results supported ***our fifth hypothesis*** and suggest that changes in microbial necromass chemistry with changing pH and interactions with different minerals regulate necromass retention and long-term persistence of MAOM.

In addition, we observed that simulated exudate chemistry influenced MAOM composition and stability only in bentonite treatments, which partly supports ***our final hypothesis***. Under the carbohydrate treatment, bentonite minerals accumulated significantly more aliphatic compounds across different pH levels, which led to significantly lower thermal stability compared with the organic acid’s treatment. Bentonite had a significantly higher respiration rate and accumulated less DOC under the carbohydrate treatment compared to the organic acid treatment. This could be due to the strong adsorption of carboxylic organic acid onto bentonite minerals through surface complexation and/or cation bridging compared with neutral carbohydrates.^39,73^ Strong adsorption decreases the substrate availability to microbes, leading to lower respiration rates and microbial activity (Figure 2a) and therefore less microbial necromass accumulation, especially aliphatic compounds (Figure 3d), and greater thermal stability compared with carbohydrate treatment (Figure 4b). These findings suggest that the effects of simulated exudate chemistry are context-dependent and mediated by mineralogy and microbial activity. However, we note that this experimental design cannot fully disentangle direct mineral–exudate sorption from microbially mediated transformation prior to mineral association because an active microbial community was present throughout the incubation. Thus, exudate-driven shifts in MAOM composition and thermal stability likely reflect a combination of (i) direct adsorption of exudate compounds that varies with mineralogy and pH and (ii) microbial uptake and conversion into microbial products/necromass that subsequently associate with minerals. We therefore interpret exudate effects conservatively as net outcomes of these concurrent pathways. Future work using abiotic (no-inoculum) controls and/or isotope labeling of exudate C would be needed to quantify the relative contributions of direct adsorption versus microbial processing across mineral–pH contexts.

Finally, we note that the simplified artificial soil approach used here is designed to isolate mineralogical and pH effects, but it does not capture all features of natural soils. In particular, field soils typically contain mixed mineral assemblages, and mineral–mineral interactions (e.g., co-occurring clays and Fe/Al oxides with contrasting surface chemistries) can modify organo-mineral binding, aggregation, and the accessibility of reactive surfaces relative to a single-mineral system. Moreover, the mineral phases used here were pristine powders and therefore lacked the weathering histories and pre-existing organo-mineral coatings that accumulate in situ; as a result, the MAOM formed during this short-term incubation should be interpreted as an early stage, operationally defined mineral-associated fraction reflecting initial microbial processing and retention on mineral surfaces, rather than a direct analog of MAOM in natural soils that emerges through decades to centuries of repeated inputs and soil-forming processes. In addition, extrapolation from this highly controlled incubation to field conditions should be made cautiously because natural soils integrate additional drivers not represented here, including plant inputs and rhizosphere processes, physical protection within aggregates, multiscale spatial heterogeneity, and temporal variability in moisture and temperature. Accordingly, our findings are best interpreted as mechanistic constraints on how pH and dominant mineral phase influence microbial processing, necromass retention, and MAOM formation, and they motivate future work that explicitly tests mixed-mineral systems, incorporates preconditioned minerals bearing natural organic coatings, and validates these mechanisms under longer-term and field conditions. Finally, mineral surface area could provide useful context for potential C coverage; however, we did not measure mineral-specific surface area (e.g., BET), and surface-area values were unavailable for all minerals. We therefore do not estimate C surface coverage to avoid uncertain calculations, and we note this as a priority for future work.

### 4.4 Implications

Soil pH has consistently been found to be one of the most important abiotic factors controlling many C-cycling processes at different scales, but its direct impact on MAOM formation and stabilization through necromass–microbe–mineral interactions has been elusive due to the environmental covariation. Here, using artificial soil systems, we isolate the independent and interactive effects of pH and mineralogy on microbial necromass retention and the thermal stability of MAOM. Our findings emphasize that microbial activity, rather than microbial carbon use efficiency, is more predictive of MAOM formation and that both necromass loading and composition (e.g., carboxyl-to-aliphatic ratio) influence MAOM persistence. These results support mechanistic zonal models of organo-mineral interaction and underscore the importance of microbial necromass chemistry in soil C sequestration. Incorporating these microbially driven, pH- and mineral-mediated stabilization mechanisms into Earth system models will improve prediction of soil C responses to land management and environmental change.

## Supplementary Material





## Notes

Full experimental details, including both the main and two complementary experiments, soil preparation, microbial culturing, pH adjustments, incubation conditions, MAOM composition, and thermal stability characterization, as well as details of methods and calculations for CUE (PDF)
Supplier-reported particle-size information, summary of three-way ANOVA results, thermogravimetry curves, carbohydrate-treatment subset structural equation model, pH value plots, soil microbial respiration dynamics, Pearson correlations, data on difference in the chemical composition, Pearson correlation matrix, correlations between MAOC and average microbial respiration rate and CUE, MIR spectra and relative abundance of each functional group, averaged relative abundance of different functional groups, data on operationally recovered coarse microbial residues, and SEM micrographs (PDF)

## Abbreviations

SOM: soil organic matter
OM: organic matter
C: carbon
MAOM: mineral-associated organic matter
MAOC: mineral-associated organic carbon
CUE: carbon use efficiency
EPS: extracellular polymer substances
WHC: water holding capacity
IRGA: infrared gas analyzer
MBC: microbial biomass carbon
MBN: microbial biomass nitrogen
DOC: dissolved organic carbon
POM: particulate organic matter
MIR: mid-infrared spectroscopy
TG: thermogravimetry
SEM: scanning electron microscopy
