# Supplementary material for: Interaction of Soil pH and Mineralogy Controls Soil Organic Matter Persistence through Changes in the Composition and Amount of Microbial Necromass
Source: Environ Sci Technol. 2026 Jul 29;60(31):21725–39. doi: 10.1021/acs.est.6c08883 (PMC13472135; doi:10.1021/acs.est.6c08883)
Supplement: Supplementary file 1 [file es6c08883_si_001.pdf]

Interaction of soil pH and mineralogy controls soil organic matter persistence through changes in the composition and amount of microbial necromass

Yuan Liu [a,b] *, Violeta Matus-Acuña [a], Lisa K Tiemann [a] *

[a] Department of Plant, Soil and Microbial Sciences, Michigan State University, East Lansing, MI, USA

[b] Physical and Life Sciences Directorate, Lawrence Livermore National Laboratory, Livermore, California, USA

* Corresponding author

E-mail address: liu95@llnl.gov (Y. Liu); ltiemann@msu.edu (L. K. Tiemann)

Full experimental details, including both main and two complementary experiments, soil preparation, microbial culturing, pH adjustments, incubation conditions, MAOM composition and thermal stability characterization, as well as details of methods and calculations for CUE are provided in the Supporting Information below.

Details of Material and Methods

1. Main experiment

1.1 Experimental design and sampling

We designed our experiment to test interactions between mineralogy and pH that influence microbial necromass accumulation as MAOM by constructing three different artificial soils, each with a different clay mineral, bentonite, kaolinite, or goethite. Each artificial soil was adjusted to four different pH levels, 5, 6, 7, and 8. As we needed a C source for microbial growth, we decided to use artificial simulated exudate cocktails for this purpose. In total, the main experiment contained 120 mesocosms with artificial soils (including 4 pH levels × 3 mineral types × 2 simulated exudate cocktails × 5 replicates). Laboratory mesocosms consisted of 100 g dry wt of artificial soil composed of 15% bentonite, kaolinite, or goethite clay minerals mixed with 85% quartz sand (Sigma-Aldrich, St. Louis, MO) inside 946 ml mason jars (Ball Corporation, USA) that could be fitted with airtight lids containing rubber septa for gas sampling. The sand consisted of a mixture of particles ranging in size from 0.074 to 1.2 mm (10% 0.074 mm, 20% 0.125 mm, 30% 0.21 mm, 25% 0.84 mm, and 15% 1.2 mm) to produce a more realistic soil particle size distribution (Qi et al., 2018). The sand was washed with Milli-Q water and combusted to remove any potential C. Before the incubation, kaolinite and goethite minerals and sand particles were added to Milli-Q water in a 1:2.5 w/v ratio, while bentonite and sand particles were mixed with Milli-Q water in a 1:5 w/v ratio due to the swelling properties of the 2:1 type clay. The pH values of the pure bentonite, kaolinite, and goethite, measured in 1:2.5 soil to water mixtures (SevenEasy S20, Mettler Toledo, OH, USA) were 9.96, 7.29, and 4.26, respectively. Artificial soils were adjusted to target pH by iterative titration using 0.1M HCl and

0.1M NaOH solutions. Exact acid/base volumes were not recorded for individual mesocosms because additions were made incrementally until the target pH was reached; start- and end-point pH measurements are reported in Fig. S3. Mineral and sand mixtures were dried to approximately 5% water holding capacity (WHC) between titration steps and pH was re-measured (1:2.5 soil:water slurry). This adjust–dry–measure cycle was repeated until the final experimental soil pH was reached (±0.1). To begin the experiment, the pH-adjusted artificial soils received microbial inoculum (~5 mL of cultured inoculum adjusted to OD ≈ 1.0 per jar) and one of the two simulated exudate cocktails at a rate of 2 mg C g^{-1} dry soil per addition event. Simulated exudate cocktails were prepared as aqueous stock solutions and added to mesocosms by pipetting aliquots of solution. Each cocktail consisted of equal amounts of each component within the cocktail: carbohydrates (glucose, fructose, arabinose) or organic acids (succinic acid, malic acid, citric acid). Carbohydrate stock solutions were sterilized by 0.22 µm filtration, while organic acid stock solutions were autoclaved prior to use. Exudate solutions were added while maintaining the target soil water content; therefore, the volume and stock concentration were adjusted as needed to keep moisture constant while delivering the prescribed total C addition. The microbial inoculum was created using soils collected from a switchgrass (*Panicum virgatum* var. Cave-In-Rock) field at 0-10 cm depth from the Kellogg Biological Station of Michigan State University. We added 0.25 g soil to a 200 ml liquid growth media (minimal media: 0.0025 M KCl, 0.0025M NaCl, 0.0005 M KH_2PO_4, 0.001 M Mg_2SO_4 and micronutrient solution: H_3BO_3, $MnCl_2$ x $4H_2O$, $CuSO_4$ x $5H_2O$, $ZnSO_4$ x $7H_2O$, Na_2MoO_4 x $2H_2O$, NaFeEDTA) containing the simulated exudates as the C source. The artificial simulated exudate cocktails were composed of mixtures of either carbohydrates (glucose, fructose, arabinose) or organic acids (citric acid, malic acid, and succinic acid) that are commonly found in switchgrass root exudates (Smercina et al.,

2019). After 2 d of growth, we used this media to then inoculate eight different growth media, containing either the carbohydrate or organic acid cocktails, adjusted to each of the four different experimental pH levels (Fig. 1b). The microbes were harvested from these eight media at stationary stage by centrifuging for 20 min at 3,000 rpm and washing three times with 1x phosphate buffered saline (PBS). The final microbial pellets were diluted with 1x PBS to an optical density of 1.0 at 600 nm. After the addition of the appropriate microbial inoculum and simulated exudate cocktail, soil moisture was brought to, and maintained at 50-65% WHC, and mesocosms were incubated at room temperature (~24° C). The two simulated exudate cocktails were added every other day at a rate of 2 mg C g^{-1} dry soil in a modified Hoagland's solution (Conn et al., 2013; Additional file 1) with a C: N ratio of 5: 1. Simulated exudates solutions were buffered to the target pH using MES buffer (2-(N-morpholino) ethane sulfonic acid; pH 5–6) or HEPES buffer (4-(2-hydroxyethyl)-1-piperazineethanesulfonic acid; pH 7–8) prior to addition. Mesocosms were incubated for 4 weeks (Fig. 1). Buffers were used only for preparing simulated exudate solutions (MES for pH 5–6; HEPES for pH 7–8); soil pH was adjusted separately by titration with 0.1 M HCl/NaOH. Buffer concentration/total buffer mass added was not recorded. To account for potential buffer-related effects on end-point MAOC, an abiotic buffer-only control (no microbial inoculation) was included. MAOC values for buffered exudate treatments were corrected by subtracting end-point MAOC measured in the corresponding abiotic buffer-only control (no inoculum). Buffer sorption/competition for mineral sorption sites was not directly quantified. Total C inputs from added simulated exudate cocktails over the 4-week incubation amounted to 26 mg C per g soil and total added inorganic N from the nutrient solution was 5.2 mg N per g soil. After 4 weeks, C cocktails and nutrient additions ceased but soils were

maintained for an additional 2 weeks under the same conditions to facilitate microbial uptake of the remaining substrate and microbial biomass turn-over and necromass accumulation.

1.2 Microbial carbon use efficiency (CUE)

Microbial carbon use efficiency (CUE) was quantified at the end of the main incubation using a post-harvest, short-term ^{13}C tracer assay that partitions added substrate C between substrate-derived microbial biomass C and substrate-derived respired CO_2-C (Geyer et al., 2019). This CUE assay was conducted in separate incubation vials after harvesting soils from the main mesocosms.

Briefly, 20 g of fresh artificial soil from each treatment was placed into a 100 ml sealed vial and amended with 1 ml of 20 atom% ^{13}C-labeled substrate solution at 100 µg C per g dry soil. Treatments received ^{13}C-glucose for the carbohydrate cocktail or ^{13}C-succinic acid for the organic acid cocktail to match the dominant C class of the simulated exudate treatment. Vials were sealed and incubated at room temperature to allow substrate uptake and respiration. Because respiration rates differed substantially at the end of the main incubation, carbohydrate treatment soils were incubated for 48 h, whereas the organic-acid treatment soils were incubated for 1 week to accumulate sufficient CO_2 for isotopic analysis.

At the end of the tracer incubation, two 12-ml headspace gas samples were withdrawn from each sealed vial; one was analyzed on a Trace 1300 gas chromatograph (Thermo Scientific, USA) to determine CO_2 concentration, and the other was analyzed by IRMS (UC Davis Stable Isotope Facility) to determine atom%$^{13}CO_2$. These data were combined to calculate cumulative substrate-derived respired C. Immediately after gas sampling, soils were processed for microbial biomass carbon (MBC) using a chloroform fumigation–extraction (CFE) approach (Tiemann and

Billings, 2011). Briefly, paired fumigated and unfumigated subsamples were extracted with 0.05 M K_2SO_4 at a 1:5 soil: extractant ratio by shaking for 1 h. DOC concentration was quantified using a Vario Select TOC/TN analyzer (Elementar, Ronkonkoma, NY, USA), and DOC isotope composition (atom%^{13}C-DOC) was determined by IRMS at the University of California, Davis Stable Isotope Facility (IRMS; Thermo Scientific, Bremen, DE).

Microbial growth from the added ^{13}C substrate (^{13}MBC) and CUE were calculated following (Geyer et al., 2019) using ^{13}C atom% in fumigated (F) and non-fumigated (NF) 0.05 M K_2SO_4 extracts. The atom% of microbial biomass C (at%MBC) was calculated as:

$$at\%\,MBC = \frac{(at\%F_{DOC} \times F_{DOC}) - (at\%NF_{DOC} \times NF_{DOC})}{(F_{DOC} - NF_{DOC})} \tag{1}$$

where F_{DOC} and NF_{DOC} are total DOC concentrations (µg C g^{-1} soil) in fumigated (F) and non-fumigated (NF) extracts, and at% F_{DOC} and at%NF_{DOC} are the corresponding ^{13}C atom% values. The percent of microbial biomass labeled by the amendment (%^{13}MBC) was calculated as:

$$\%\,^{13}MBC = \frac{at\%MBC_t - at\%\,MBC_{ctrl}}{at\%_{substrate} - at\%MBC_{ctrl}} \times 100 \tag{2}$$

where $at\%MBC_t$ is *at%MBC* for the labeled treatment, $at\%MBC_{ctrl}$ is *at%MBC* for the corresponding unlabeled (natural abundance) substrate control processed identically, and $at\%_{substrate}$ is the atom% of the amendment solution (20 atom%).

Total microbial biomass C (MBC; µg C g^{-1} soil) was calculated by chloroform fumigation–extraction as:

$$MBC = (F_{DOC} - NF_{DOC})/k_{EC} \tag{3}$$

where k_{EC} = 0.45 (Fang et al., 2022). Microbial growth derived from the added substrate (^{13}MBC; μg C g^{-1} soil) was then calculated as:

$$^{13}MBC = MBC \times (\%\ ^{13}MBC/100) \quad (4)$$

Microbial CUE was then calculated as:

$$\text{CUE} = \frac{^{13}MBC}{^{13}MBC + {}^{13}R} \quad (5)$$

where ^{13}R is the cumulative respiration derived from added substrate (μg $^{13}CO_2$–C g^{-1} soil) over the tracer incubation period.

For the organic-acid treatments, extractable DOC concentrations were insufficient to robustly quantify substrate-derived MBC across treatments; therefore, we report MBC and CUE only for the carbohydrate treatments.

1.3 Artificial mineral associated organic matter (MAOM) characterization

Here we focus on MAOM because this fraction of total SOM is most critical in determining the persistence of SOM. In brief, after incubation, 10 g of artificial soil from each treatment was mixed with 30 ml Milli-Q water in a 50 ml centrifuge tube and shaken for 18 h at 250 rpm to disperse the soil slurry. The suspension was then wet-sieved through a 53-μm mesh sieve to separate the fine mineral fraction from the quartz sand particles. Material passing the sieve (<53 μm) was operationally defined as the clay mineral–associated fraction (MAOM). The <53 μm fraction collected in the receiving vessel was washed five times with Milli-Q water to remove dissolved organic carbon (DOC) by resuspending and centrifuging at 2,000 rpm for 20 min between washes (supernatant discarded each time). Because DOC in wash supernatants was not quantified, our MAOM measurements represent the washed <53 μm fraction and do not

include the dissolved/weakly associated C removed during washing. Material retained on the 53 μm sieve (>53 μm) consisted primarily of quartz sand plus coarse organic residues formed during incubation. To quantify coarse microbial residue material independent of sand mass, the >53 μm fraction was transferred to a separate container and repeatedly rinsed with Milli-Q water; the decanted suspension containing suspended residues was passed through a 53 μm mesh sieve to capture the residue material. The collected residue fraction (hereafter referred to as the coarse microbial residue fraction) was dried at 60 °C for 48 h prior to weighing. The separated clay minerals were dried at 60°C for 48 h and ground with a ball mill to aid in homogenization for the following analyses. Mid-infrared spectroscopy (MIR) and thermogravimetric (TG) measurements were performed on these dried, homogenized powders, which minimizes heterogeneity and variability associated with local differences in organic coating or sample packing within the MIR sampling volume. MAOM composition was characterized using MIR. Specifically, ground MAOM samples were each scanned using MIR on samples compressed into aluminum microplate wells in an HTS-XT microplate reader attached to a Bruker Vertex 70 (Bruker Optics, Billerica, MA USA). We acquired spectra from 4000 to 400 cm^{-1} at 4 cm^{-1} (wavenumber) resolution (average of 32 scans). To account for the potential interference of the different minerals, we subtracted the corresponding pure mineral spectra from the sample spectra. Based on the relative absorption spectra and previous research (Parikh et al., 2014; Fang et al., 2021; Mainka et al., 2022), we differentiated three functional groups: aliphatic C-H bonds, carboxyl C=O bonds, and aromatic C=C (Fig. S3) as follows: bands at 2920 and 2850 cm^{-1} were attributed to antisymmetric and symmetric stretching vibration of C-H from aliphatic -CH_3 and -CH_2- groups (Parikh et al., 2014; Mainka et al., 2022); bands at 1620 cm^{-1} (1720-1600 cm^{-1}) represent a carboxylic C=O stretch of amides, quinones, and ketones (Parikh et al., 2014); and

bands located at 1512 cm^{-1} (1580-1490 cm^{-1}) were attributed to the stretching vibration of C=C from aromatic compounds (Parikh et al., 2014). Given that aromatic compounds are more recalcitrant than aliphatic compounds, we also calculated the ratio of peaks at 1512 cm^{-1} and the sum of peak values at 2920 cm^{-1} and 2850 cm^{-1} as a MAOM recalcitrance index (aromatic/aliphatic).

1.4 C content and thermal stability of MAOM

C content in MAOM (MAOC) was measured via combustion on a total C analyzer (LECO 832, LECO Corp., St. Joseph, MI). The thermal stability of the MAOM was investigated using thermogravimetric (TG) analysis using a TGA/DSC 1 STAR system thermogravimetric analyzer (Mettler Toledo, Columbus, Ohio, USA). For the TG analysis, dried and ground soil samples (~10 mg) were weighed out into a 150 ml aluminum oxide crucible and heated from 60 to 800 °C at a heating rate of 20 °C/min under an N_2 atmosphere (20 ml/min flow rate). The weight loss of samples was measured and reported as a function of temperature (Fig. S1). We calculated the temperature at which half of the mass was lost during TG analysis from 190 to 800°C (TG-T_{50}) to represent the thermal stability of MAOM from each artificial soil sample (Plante et al., 2011).

2. Complementary necromass-mineral association experiment

We conducted two complementary experiments in which we added artificially generated necromass to pure clay minerals to determine how differences in pH and microbial community structure would influence MAOM formation from necromass. In our first experiment, we chose to create necromass from pure cultures of three broadly defined, microbial groups – Gram-

positive and Gram-negative bacteria and fungi – that would generate chemically distinct necromass. Specifically, we chose *Bacillus cereus* (BGSC 6A1), *Pseudomonas fluorescens* (ATCC 13525), and *Schizophyllum commune* (ATCC 18246), a Basidiomycete fungus, as our representative microbes. The two bacteria were grown in Luria broth (LB) at 23°C on a rotary shaker (150 rpm) to early stationary (24 h) growth phase, then centrifuged for 20 min at 3,000 rpm, the growth medium decanted, and the cell pellet rinsed three times with sterile deionized water. The fungi were grown on solid LB media for 10 d, and all hyphae and biomass were harvested by scraping the plates. This fungal material was added to Milli-Q water, and then sonicated, which removed agar from hyphal surfaces and allowed us to separate hyphae from agar via density fractionation in water. Hyphae were rinsed three times with Milli-Q water and both bacterial cells and fungal hyphae were frozen at -80 °C, lyophilized, and ground with a ball mill. We produced 5-6 g of dry necromass for each microbe for use in the following two experiments. First, we added each of the three different types of pure microbial necromass to each of the three clay minerals that had all been adjusted to the same four pH levels as in the main experiment (Fig. 1c). Specifically, to 3 g dry weight of each mineral (kaolinite, bentonite, goethite) adjusted to pH 5, 6, 7, or 8, we added 0.3 g necromass (0.1 g necromass g^{-1} mineral or ~5% necromass-C g^{-1} mineral). The clay minerals were mixed with water to adjust pH as described above and were at a 2:1 water-to-mineral ratio before the dry necromass was added. The minerals and necromass were well mixed by hand and then samples were placed on a rotary shaker set at 200 rpm for 24 h. After shaking, samples were centrifuged at 3000 rpm for 20 min, the supernatant was removed. The mineral plus necromass pellet was resuspended with Milli-Q water, vortexed, and centrifuged three times to remove any loosely associated necromass. We interpret the remaining solid fraction as mineral-retained microbial-derived material under

standardized operational washing conditions, rather than as a direct measure of irreversible mineral binding or long-term field-scale MAOM persistence. Finally, the minerals with associated necromass were dried in an oven at 65 °C for 48 h, ground with a ball mill, and then chemical composition and thermal stability were determined using MIR spectroscopy and TG analyses as described above. We also analyzed the pure culture, dried necromass alone via MIR spectroscopy.

In a second experiment designed as a controlled loading test of microbial-derived material on mineral surfaces, *B. cereus* necromass (oven-dried at 60 °C) was added at 0, 0.01, 0.05, 0.1, or 0.2 g of necromass material to 1 g bentonite in 10 ml Milli-Q water (no pH adjustment). Necromass and minerals were hand-mixed to disperse solids and then shaken to promote mineral-organic contact. Suspensions were subsequently centrifuged, and the recovered solids were dried and ground as described above prior to MIR and TG analyses. Because this loading experiment was designed to test how increasing microbial-derived material alters mineral-associated chemical and thermal signatures under standardized preparation conditions, results are interpreted as a relative comparison across loading levels rather than a quantitative measure of long-term stabilization. TG analyses were conducted on ~10 mg of dried, homogenized material per run and thermal stability was summarized using TG-T_{50} (temperature at which 50% of total mass loss occurred), a mass-normalized index used for comparative interpretation across loading levels.

References

Fang, K., Chen, L., Qin, S., Zhang, Q., Liu, X., Chen, P., Yang, Y., 2021. Mineral and Climatic Controls Over Soil Organic Matter Stability Across the Tibetan Alpine Permafrost Region. Global Biogeochemical Cycles 35, 1–12. doi:10.1029/2021GB007118

Fang, Y., Tavakkoli, E., Weng, Z., Collins, D., Harvey, D., Karimian, N., Luo, Y., Mehra, P., Rose, M.T., Wilhelm, N., Zwieten, L. Van, 2022. Disentangling carbon stabilization in a Calcisol subsoil amended with iron oxyhydroxides : A dual- 13 C isotope approach. Soil Biology and Biochemistry 170, 108711. doi:10.1016/j.soilbio.2022.108711

Geyer, K.M., Dijkstra, P., Sinsabaugh, R., Frey, S.D., 2019. Clarifying the interpretation of carbon use efficiency in soil through methods comparison. Soil Biology and Biochemistry 128, 79–88. doi:10.1016/j.soilbio.2018.09.036

Jenkinson, D.S., Brookes, P.C., Powlson, D.S., 2004. Measuring soil microbial biomass. Soil Biology and Biochemistry 36, 5–7. doi:10.1016/j.soilbio.2003.10.002

Mainka, M., Summerauer, L., Wasner, D., Garland, G., Griepentrog, M., Berhe, A.A., Doetterl, S., 2022. Soil geochemistry as a driver of soil organic matter composition : insights from a soil chronosequence. Biogeosciences 19, 1675–1689.

Parikh, S.J., Goyne, K.W., Margenot, A.J., Mukome, N.D., Calderón, F.J., 2014. Soil chemical insights provided through vibrational spectroscopy, Advances in agronomy. Elsevier. doi:10.1016/B978-0-12-800132-5.00001-8

Plante, A.F., Fernández, J.M., Haddix, M.L., Steinweg, J.M., Conant, R.T., 2011. Biological, chemical and thermal indices of soil organic matter stability in four grassland soils. Soil

Biology and Biochemistry 43, 1051–1058. doi:10.1016/j.soilbio.2011.01.024

Qi, F., Zhang, R., Liu, X., Niu, Y., Zhang, H., Li, H., Li, J., Wang, B., Zhang, G., 2018. Soil particle size distribution characteristics of di ff erent land-use types in the Funiu mountainous region. Soil & Tillage Research 184, 45–51. doi:10.1016/j.still.2018.06.011

Tiemann, L.K., Billings, S.A., 2011. Changes in variability of soil moisture alter microbial community C and N resource use. Soil Biology and Biochemistry 43, 1837–1847. doi:10.1016/j.soilbio.2011.04.020

Vance, E.D., Brookes, P.C., Jenkinson, D.S., 1987. An extraction method for measuring soil microbial biomass C. Soil Eiol. Biochem. 19, 703–707.
