# Supplementary material for: Interaction of Soil pH and Mineralogy Controls Soil Organic Matter Persistence through Changes in the Composition and Amount of Microbial Necromass
Source: Environ Sci Technol. 2026 Jul 29;60(31):21725–39. doi: 10.1021/acs.est.6c08883 (PMC13472135; doi:10.1021/acs.est.6c08883)
Supplement: Supplementary file 2 [file es6c08883_si_002.pdf]

Interaction of soil pH and mineralogy controls soil organic matter persistence through changes in the composition and amount of microbial necromass

Yuan Liu [a,b]*, Violeta Matus-Acuña [a], Lisa K Tiemann [a]*

[a] Department of Plant, Soil and Microbial Sciences, Michigan State University, East Lansing, MI, USA

[b] Physical and Life Sciences Directorate, Lawrence Livermore National Laboratory, Livermore, California, USA

* Corresponding author

E-mail address: liu95@llnl.gov (Y. Liu); ltiemann@msu.edu (L. K. Tiemann)

Supplementary Table S1-S2 and Fig S1-18

Table S1. Supplier-reported particle-size information for mineral powders used in the artificial soil system and literature-reported size information where available. Supplier particle-size information is as provided by Sigma-Aldrich and may be historical (not necessarily tested lot-to-lot). For goethite (Sigma-Aldrich 71063), the supplier does not report particle size ("not determined"); therefore, a published size distribution for the same SKU is provided as a reference.

Mineral	Supplier (Product No.)	Supplier- and/ or literature-reported particle size information	Notes
Kaolinite	Sigma-Aldrich (03584)	Historical cumulative distribution: ~99% < 20 µm; ~90% < 10 µm; ~75% < 6 µm; ~60% < 4 µm; ~40% < 2 µm	Historical values; not necessarily tested lot-to-lot
Bentonite (montmorillonite-rich)	Sigma-Aldrich (285234)	Historical metrics: 100% < 74 µm; 99.75% < 44 µm; mean particle size = 5.45 µm	Historical values; not necessarily tested lot-to-lot
Goethite	Sigma-Aldrich (71063; "Goethite, 30–63% Fe")	Mean particle size of 411.8 nm (range 164.2–955.4 nm)	Supplier does not report PSD for this product (not determined); published size distribution for the same SKU is reported in Fig. S1 of Liu et al., 2020 as a reference

Note: Sigma-Aldrich does not provide complete lot-specific exchangeable cation chemistry (e.g., Ca^{2+}, Mg^{2+}, Na^{+}, CEC) for the clay minerals used here, and exchangeable cations were not measured in this study. For bentonite (Sigma-Aldrich 285234), Sigma technical support indicates the product is not supplied as a calcium or sodium form (CAS 1302-78-9).

Reference: Liu, G., Zhang, Y., Yu, H., Jin, R., & Zhou, J. (2020). Acceleration of goethite-catalyzed Fenton-like oxidation of ofloxacin by biochar. Journal of Hazardous Materials, 397, 122783.

Table S2 Summary (F-values) of three way-ANOVA for the effects of mineral, necromass type, pH treatment, and their interactions on the composition and thermal stability of mineral-associated necromass. *, **, *** represent significance at $P < 0.05$, $P < 0.01$, and $P < 0.001$, respectively.

Variables	Mineral	Necromass	pH	Mineral * Necromass	Mineral * pH	Necromass * pH	Mineral * Necromass * pH
Aliphatic	7270.92***	2.32	8.61***	18.06***	22.82***	5.04***	8.40
Carboxyl	276.23***	169.34***	4.29**	27.53***	3.82**	0.60	1.91
Aromatic	364.33***	324.09***	1.7	22.78***	4.92***	2.80*	2.27*
TGA	9127.02***	109.97***	0.54	56.48***	0.62	0.40	1.02

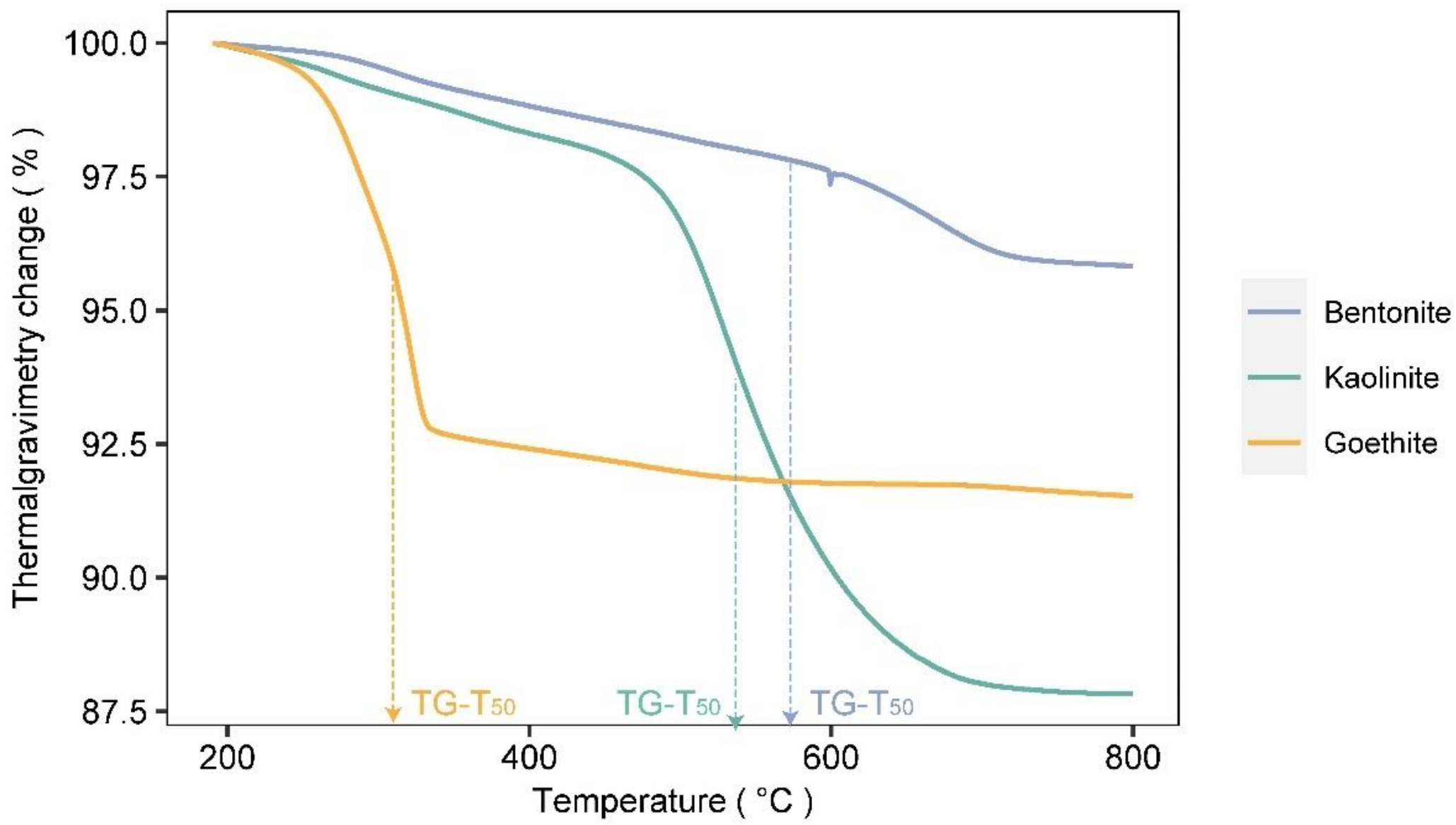

Fig. S1 Thermogravimetry (TG) curves of different minerals and estimated thermal stability indices (TG-T_{50}). Curves were selected from three minerals at pH=5 under carbohydrate treatment as an example.

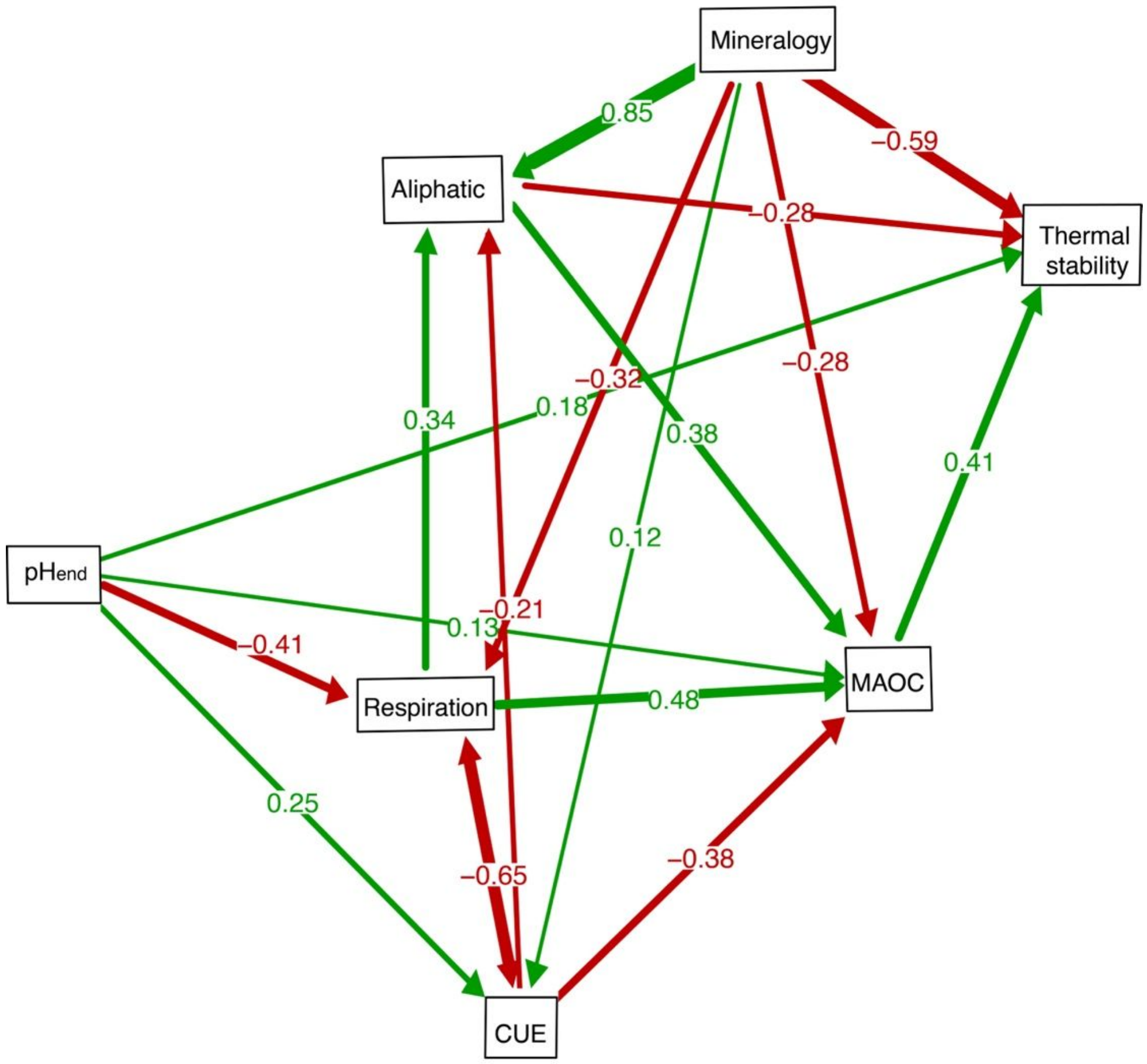

Fig. S2 Carbohydrate-treatment subset structural equation model (SEM) including carbon use efficiency (CUE). The subset SEM was conducted because CUE was quantifiable only for the carbohydrate treatments and not for the organic-acid treatments. The model evaluates pathways linking end-point pH (pHend) and mineralogy to MAOC and thermal stability via respiration (microbial processing intensity), CUE, and an MIR-derived aliphatic index. Values on arrows are standardized path coefficients; green and red arrows indicate positive and negative effects, respectively, with line width proportional to effect magnitude. Model fit indices were mixed and are reported for transparency: $\chi^2 = 17.18$, df = 3, $p = 0.001$, CFI = 0.954, RMSEA = 0.324, SRMR = 0.020. This model is presented as a sensitivity analysis and should be interpreted conservatively.

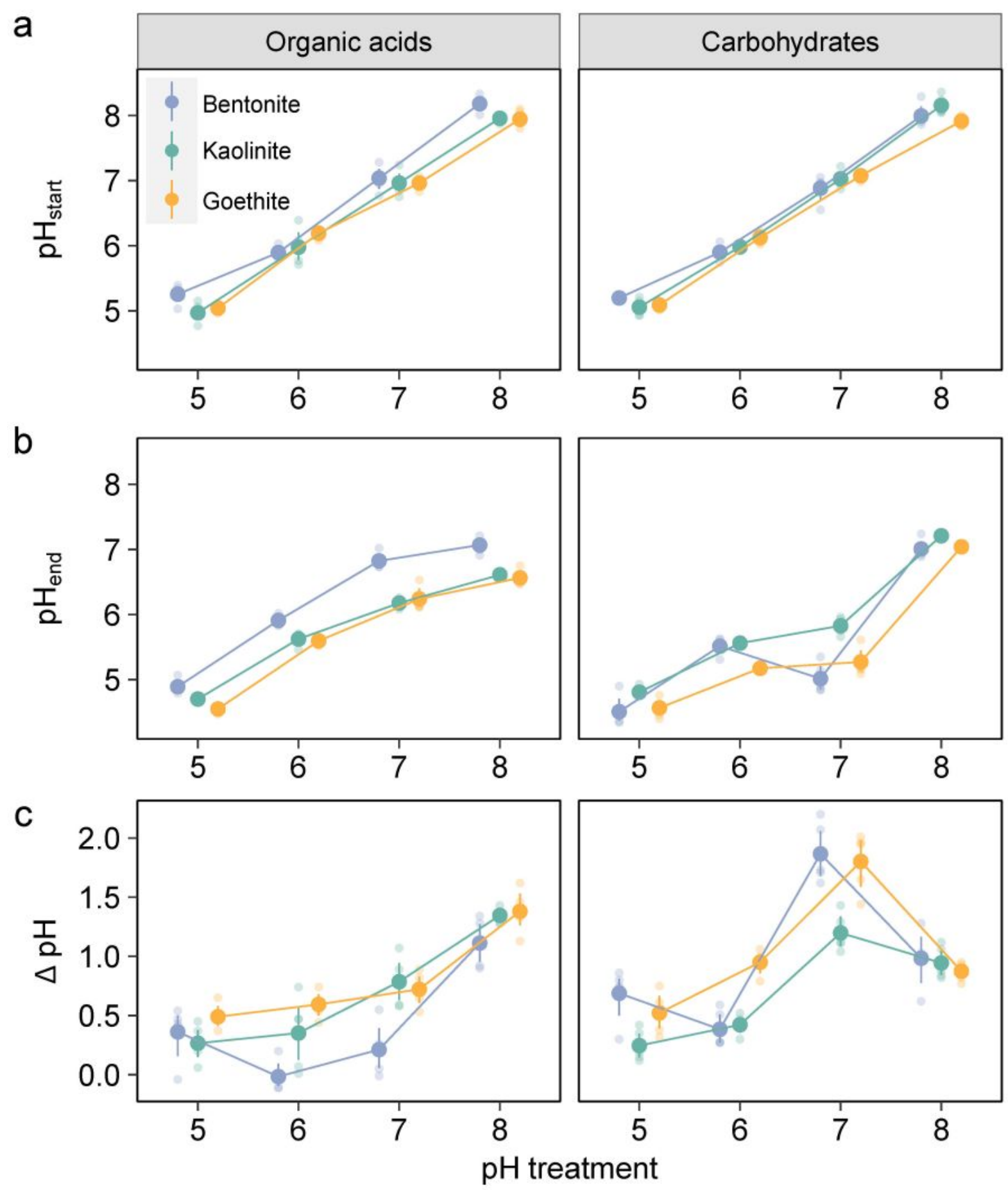

Fig. S3 pH values at the beginning (a), the end (b), and the difference between beginning and the end (c) of incubation of three different minerals at four pH levels with two root exudates addition. Data are mean values ± se (n=5).

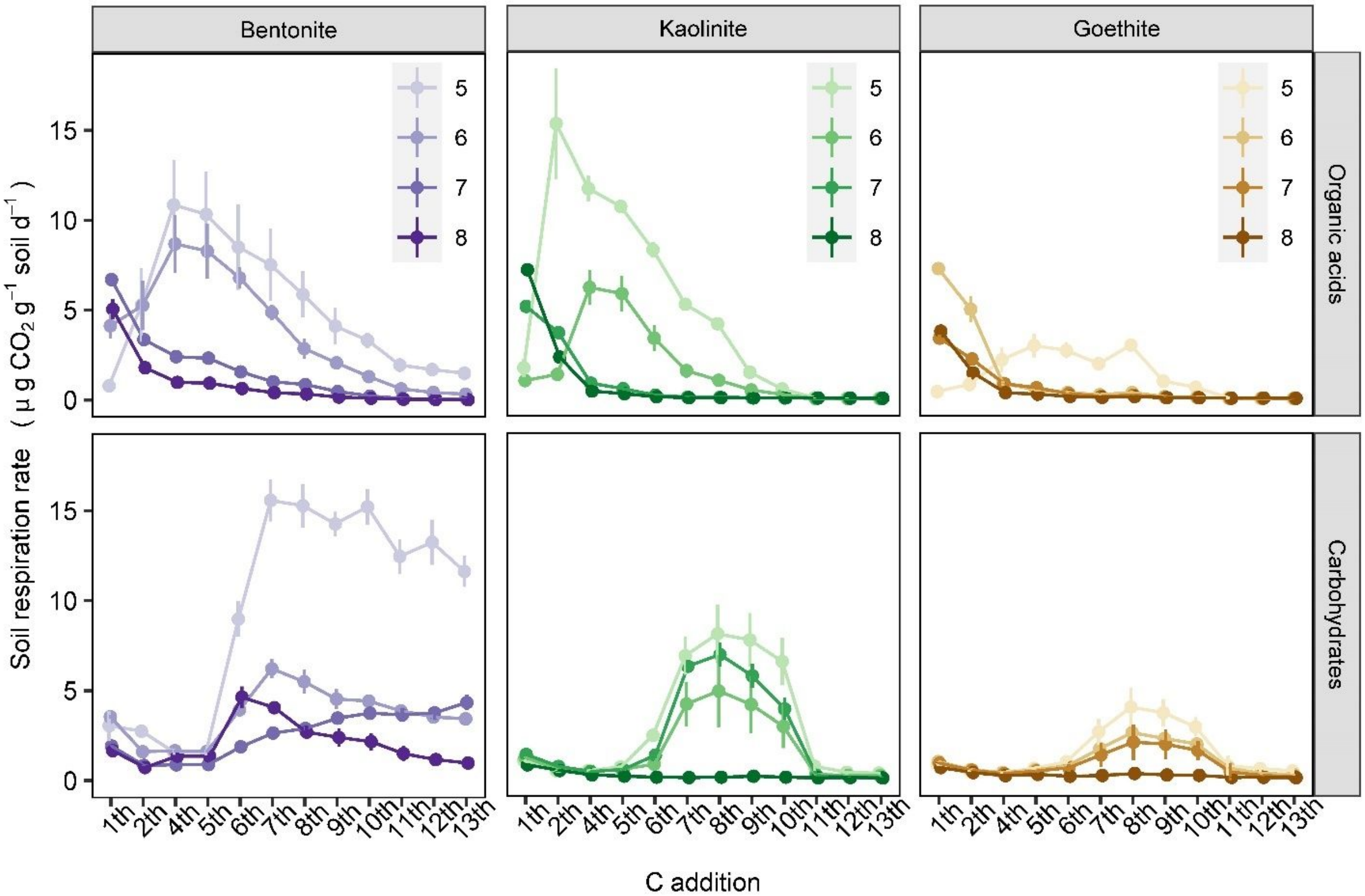

Fig. S4 Soil microbial respiration dynamics followed by each C addition over 6 weeks incubation in different minerals at different pH levels with two root exudate treatments. Data are mean values ± se (n=5).

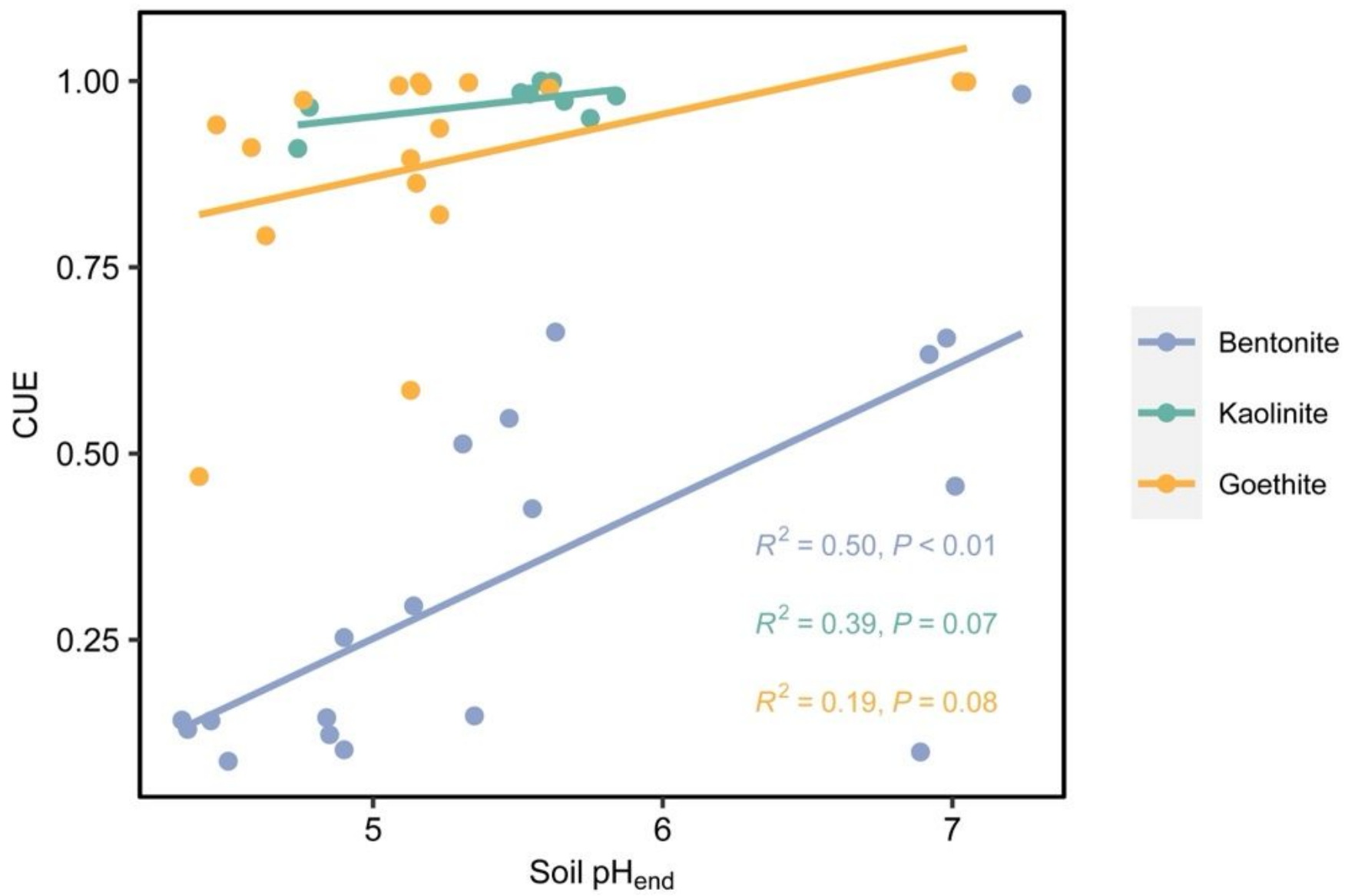

Fig. S5 Pearson correlations between microbial carbon use efficiency (CUE) and soil pH at the end of incubation (pH_{end}).

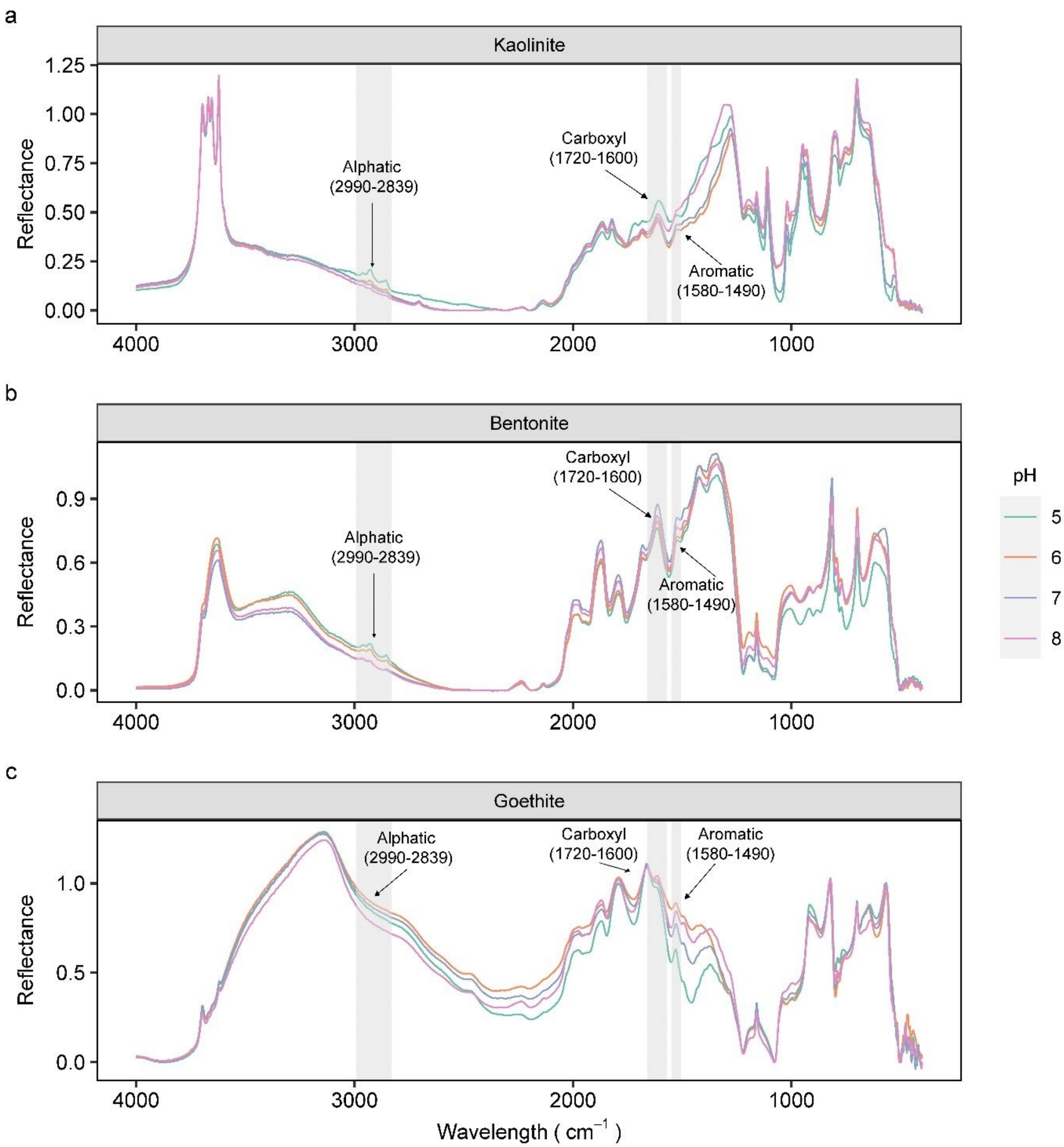

Fig. S6 Difference in the chemical composition of mineral-associated OM fraction (MAOM) from the main incubation experiment. Mid-infra-red (MIR) spectra displaying the assigned peaks of different functional groups of MAOM at different soil pH levels in organic acids treatment as an example.

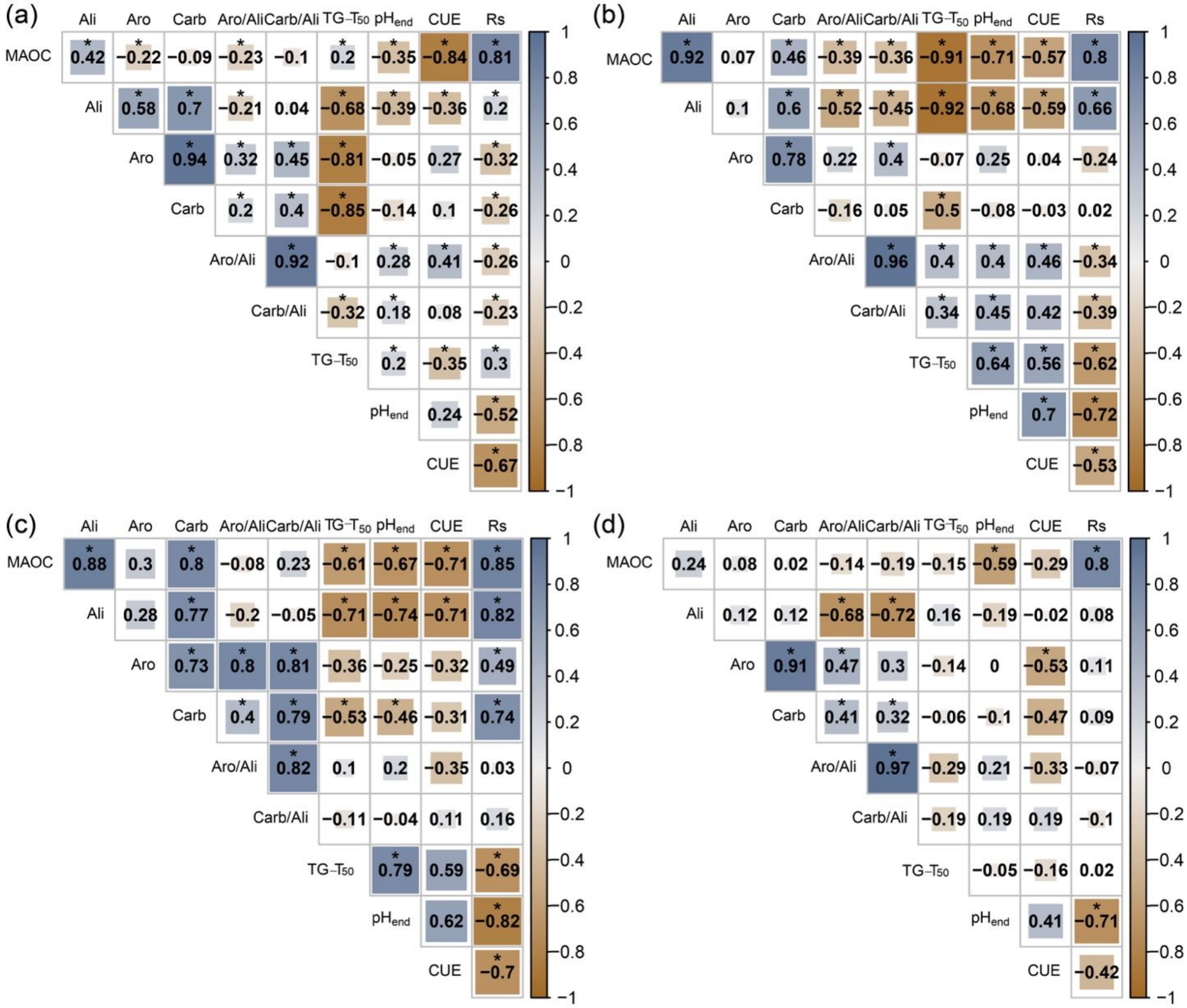

Fig. S7. Pearson correlation matrix between the content, composition, and stability of mineral-associated organic matter (MAOM) and pH_{end}, carbon use efficiency (CUE), and microbial activity (Rs, average respiration rate) for all the minerals (a), bentonite (b), kaolinite (c), and goethite (d), respectively. MAOC: mineral-associated organic carbon content; Ali: aliphatic compounds; Aro: aromatic compounds; Carb: carboxyl compounds; Aro/Ali: ratio of aromatic and aliphatic compounds; Carb/Ali: ratio of carboxyl and aliphatic compounds. TG-T_{50}: thermal stability indices. * indicate significant correlations at $P < 0.05$ level.

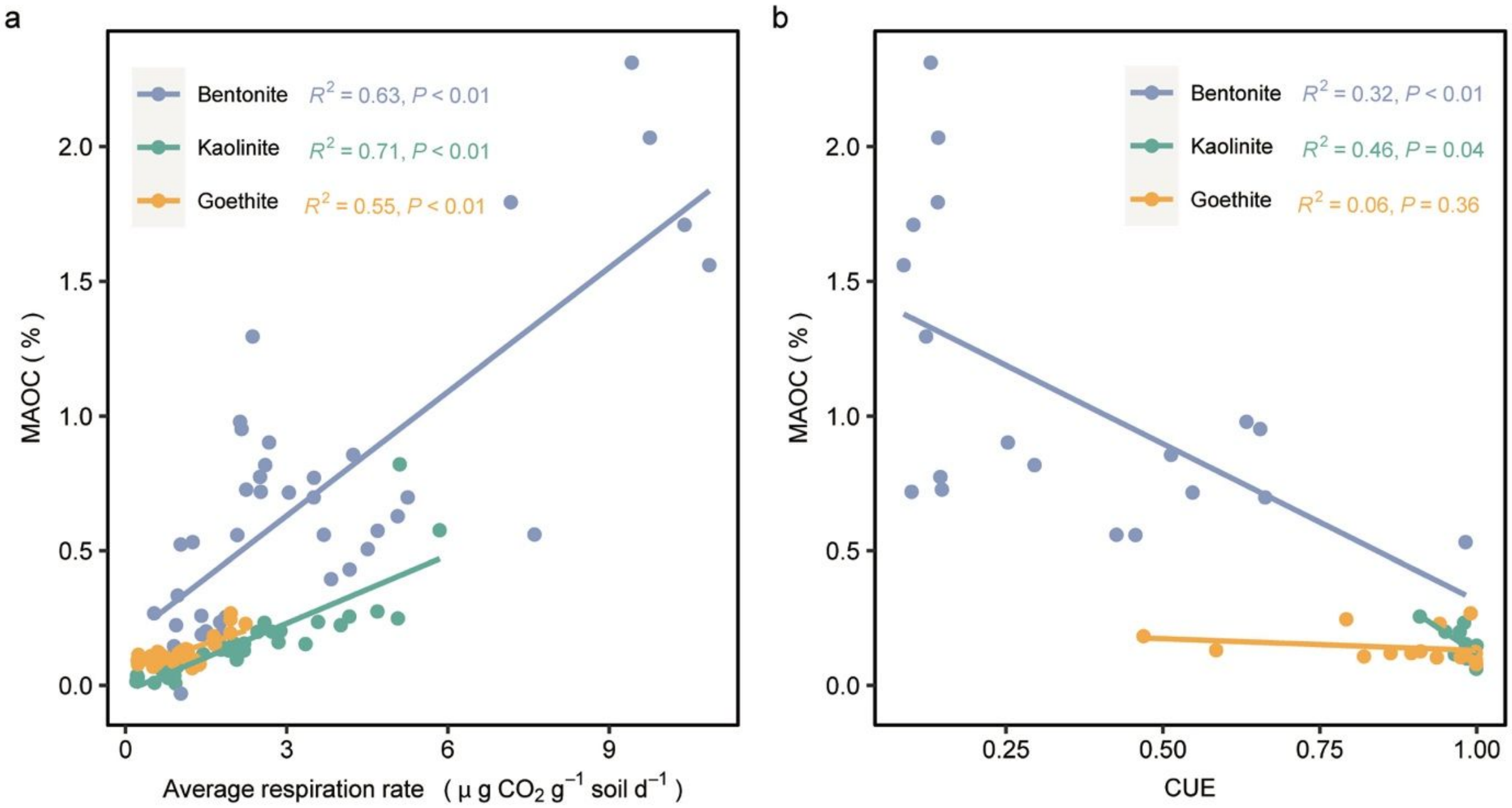

Fig. S8 Correlations between mineral-associated organic carbon (MAOC) with average microbial respiration rate (a) and carbon use efficiency (CUE, b).

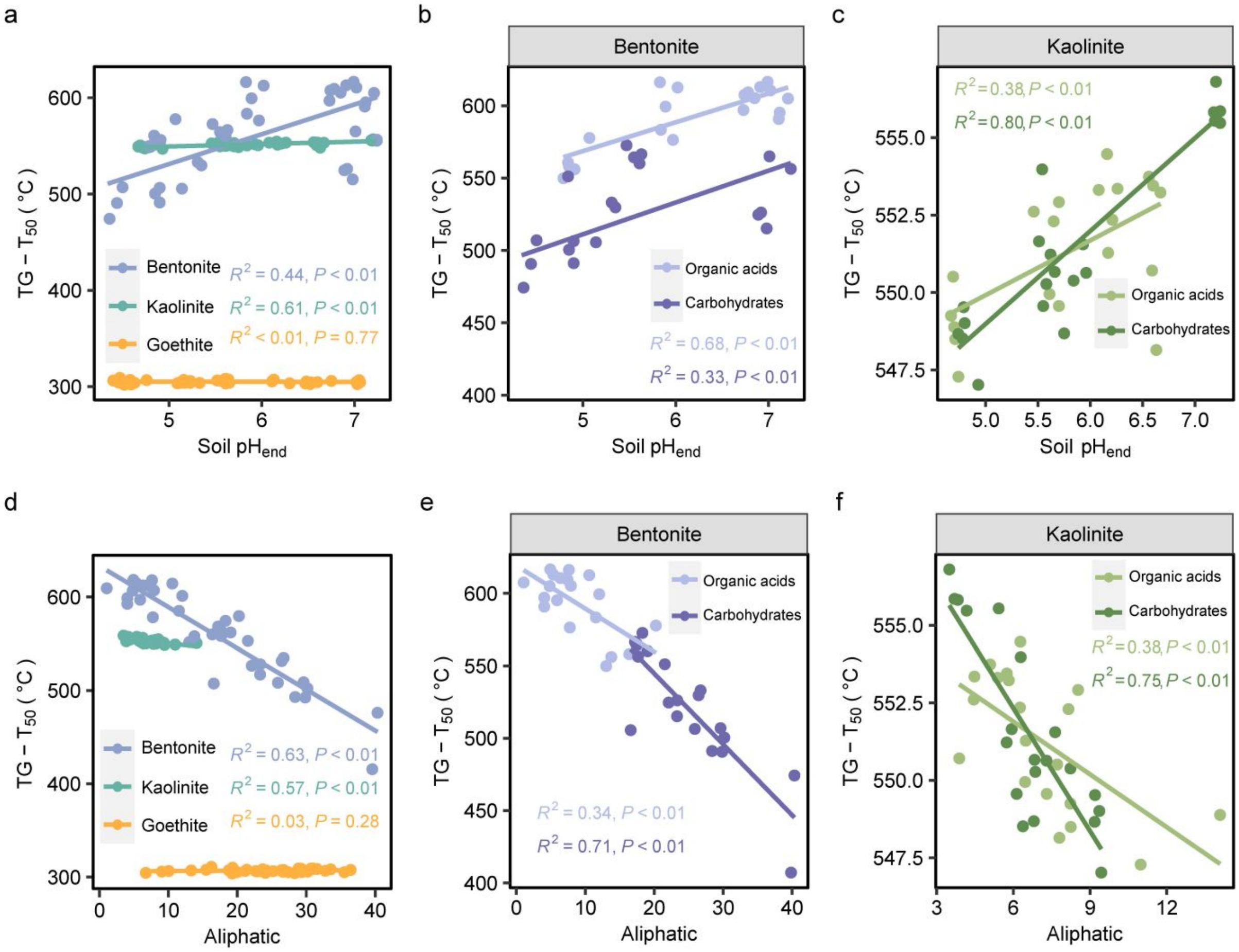

Fig. S9 Pearson correlations between thermal stability indices (TG-T_{50}) of mineral-associated organic matter (MAOM) with soil pH at the end of incubation (pH_{end}) and aliphatic compounds of MAOM across different pH and root exudate treatments.

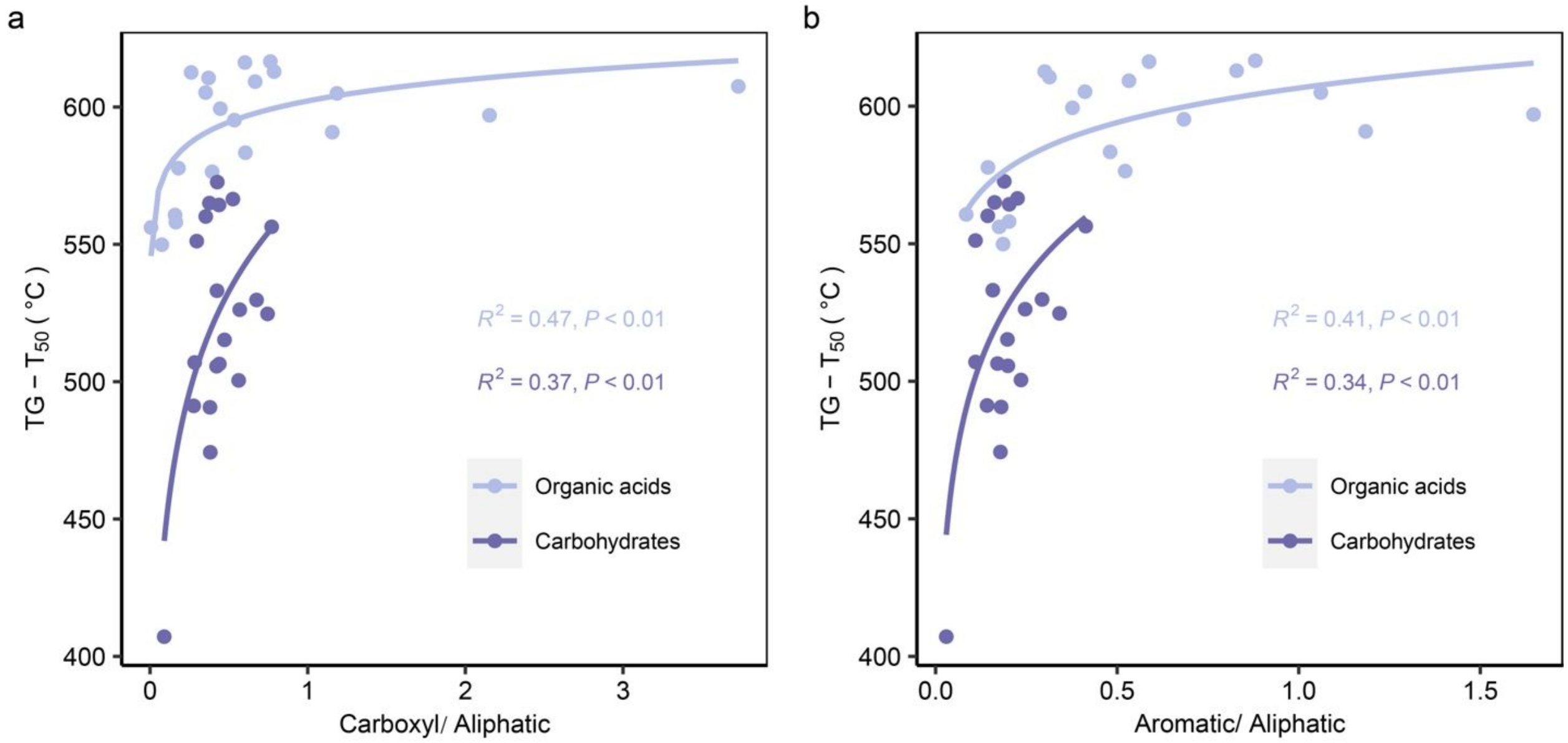

Fig. S10 Pearson correlations between thermal stability indices (TG-T_{50}) of mineral-associated organic matter with the ratio of carboxyl and aliphatic compounds and the ratio of aromatic and aliphatic compounds for bentonite mineral for two exudate treatments.

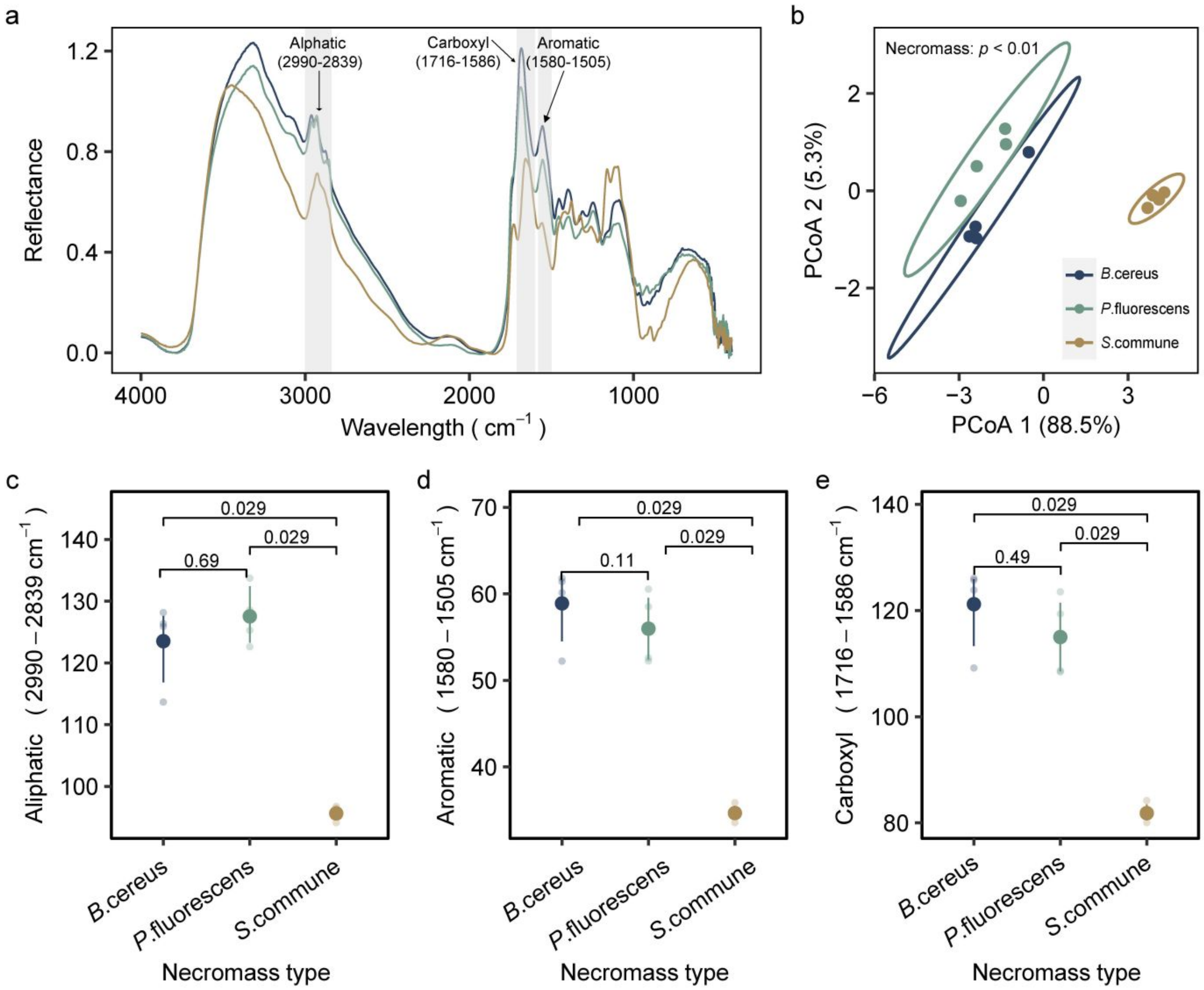

Fig. S11 Mid infrared spectroscopy (MIR) spectra and assigned peak of functional groups (a), the PCoA analysis of the whole MIR spectrum for the microbial necromass of the three pure species (b), and the relative abundance of each functional group for the microbial necromass of the three pure species (c). Data are mean values ± se (n=3).

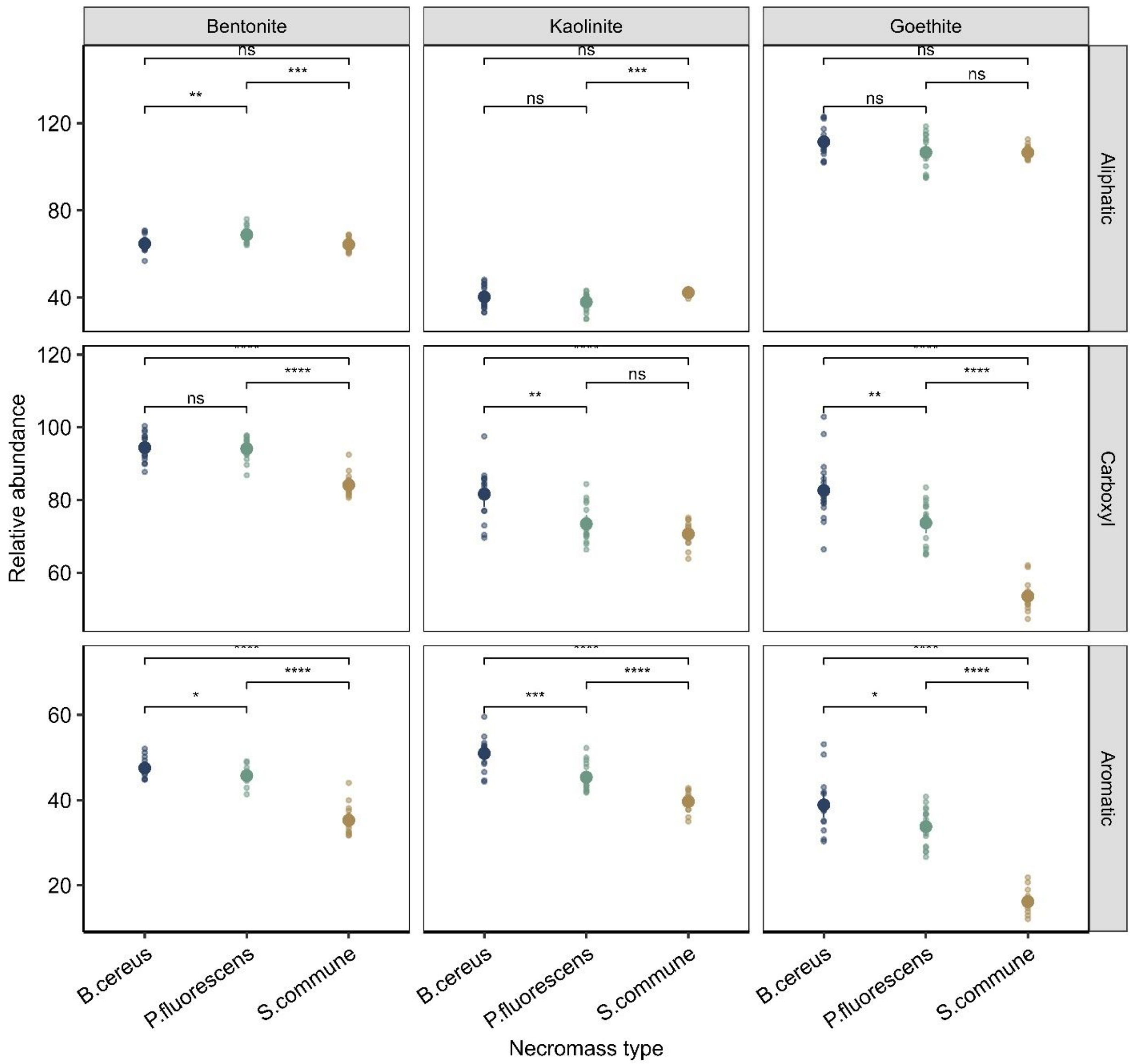

Fig. S12 The averaged relative abundance of different functional groups from the necromass of three pure species associated with different minerals examined by MIR. ns, *, **, *** represent no significance, and significance at $P < 0.05$, $P < 0.01$ and $P < 0.001$, respectively.

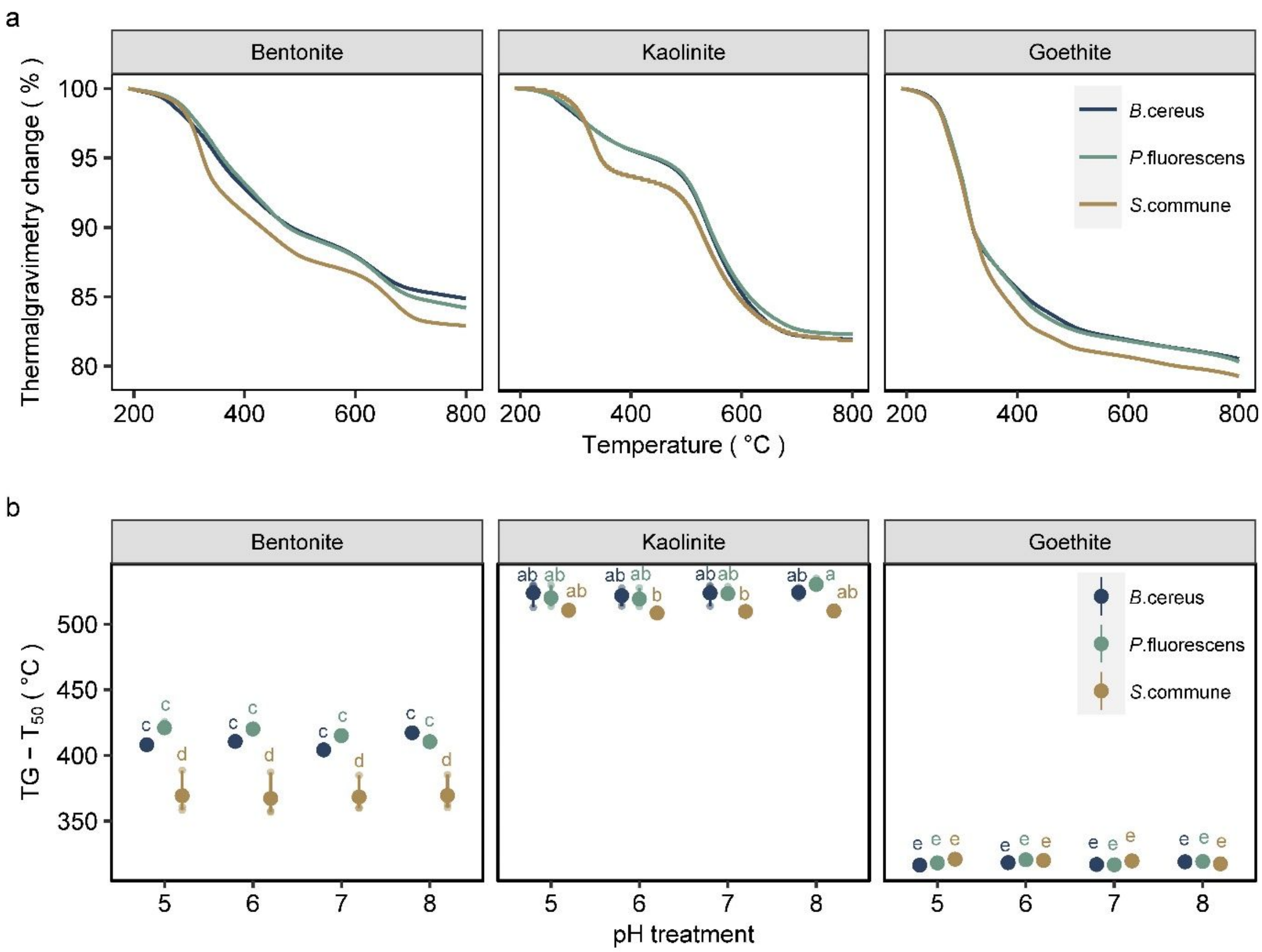

Fig. S13 Thermogravimetry (TG) curves (a) and thermal stability indices (TG-T_{50}) of different microbial necromass across different minerals at different pH levels. Lower case letters indicate significant differences among different necromass types, pH levels and mineral types. Data are mean values ± se (n=3).

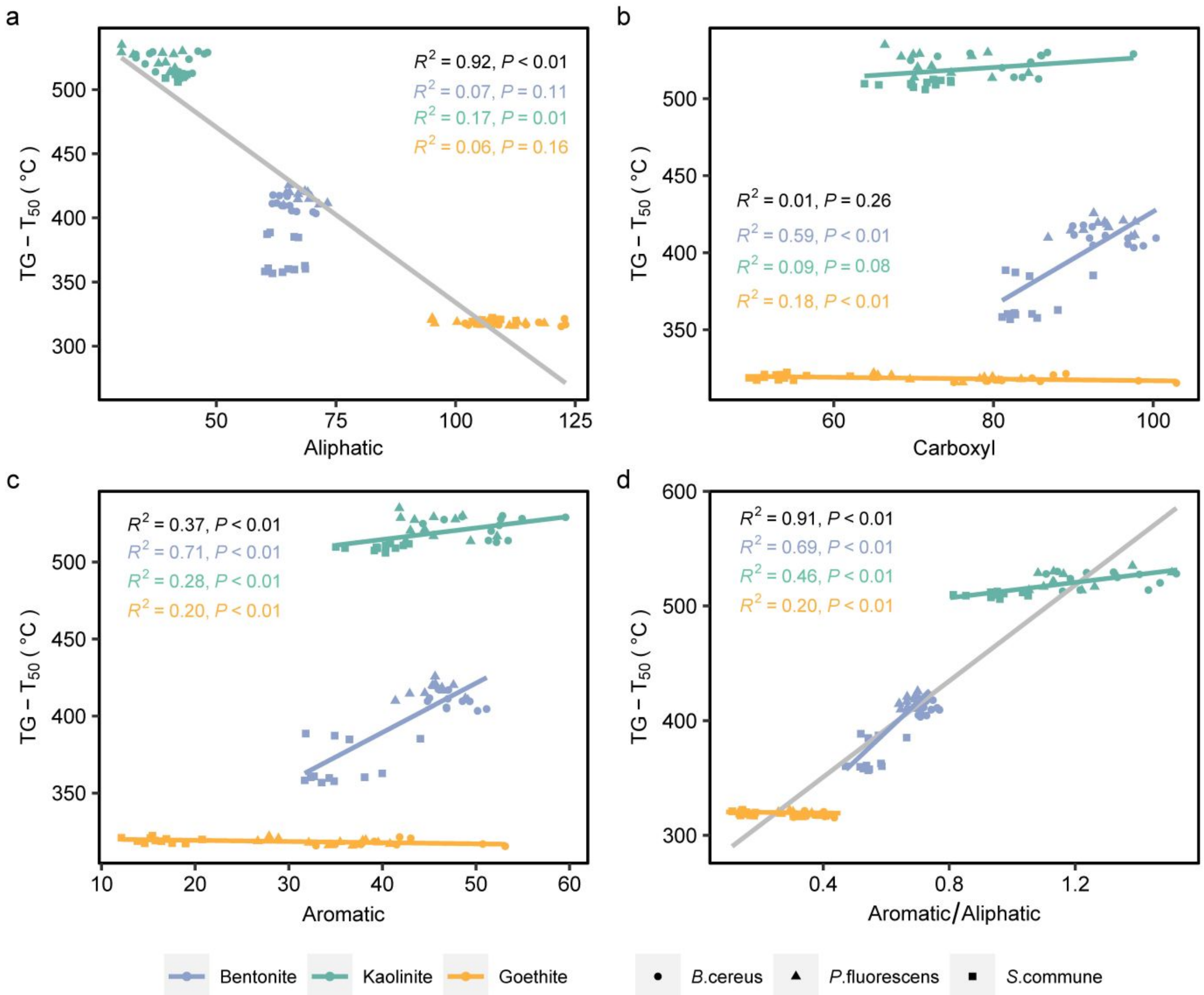

Fig. S14 Pearson correlations between the thermal stability indices (TG-T_{50}) of mineral-associated necromass with the relative abundance of functional group of MAOM across different minerals and necromass types.

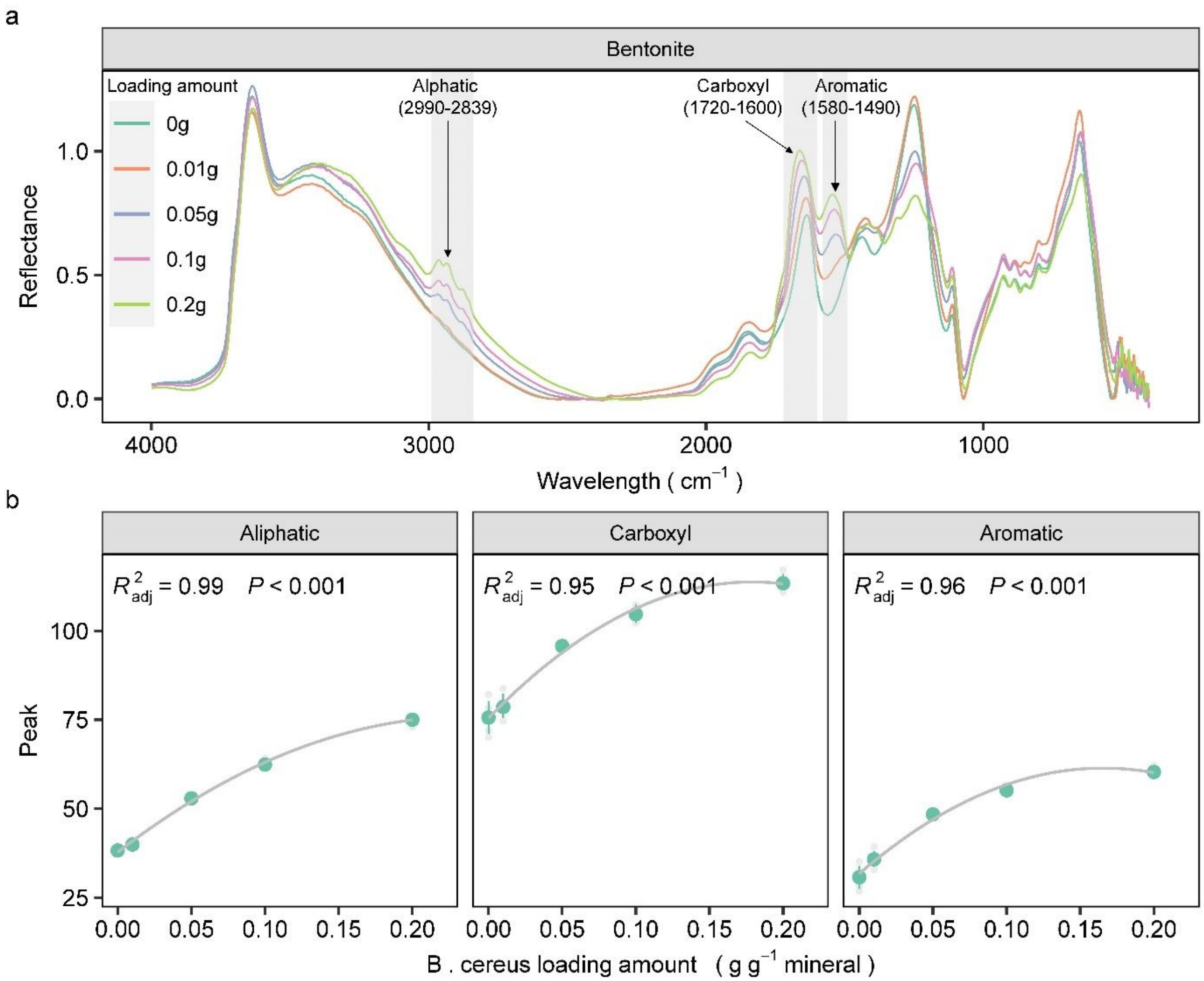

Fig. S15 MIR spectra and assign peak of different functional group at different loading amount of the necromass from the gram- positive bacteria (*B*. cereus) associated with bentonite (a) and response curve of the relative abundance of different functional groups of mineral-associated necromass (b) with loading amount of necromass.

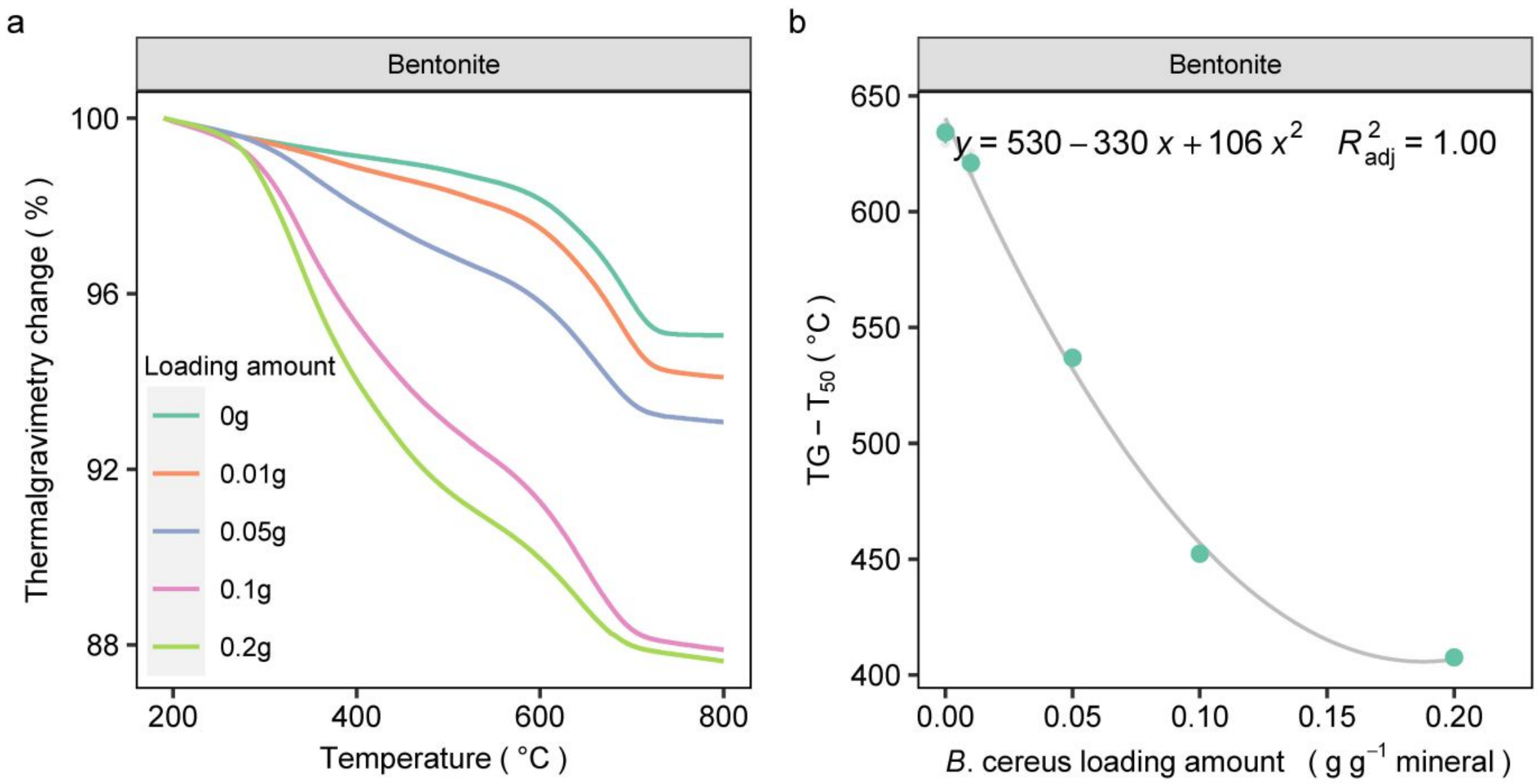

Fig. S16 Thermogravimetry (TG) curves (a) and thermal stability indices (TG-T_{50}, b) of mineral-associated organic matter in bentonite mineral receiving different amounts of gram-positive bacteria (*B*. cereus).

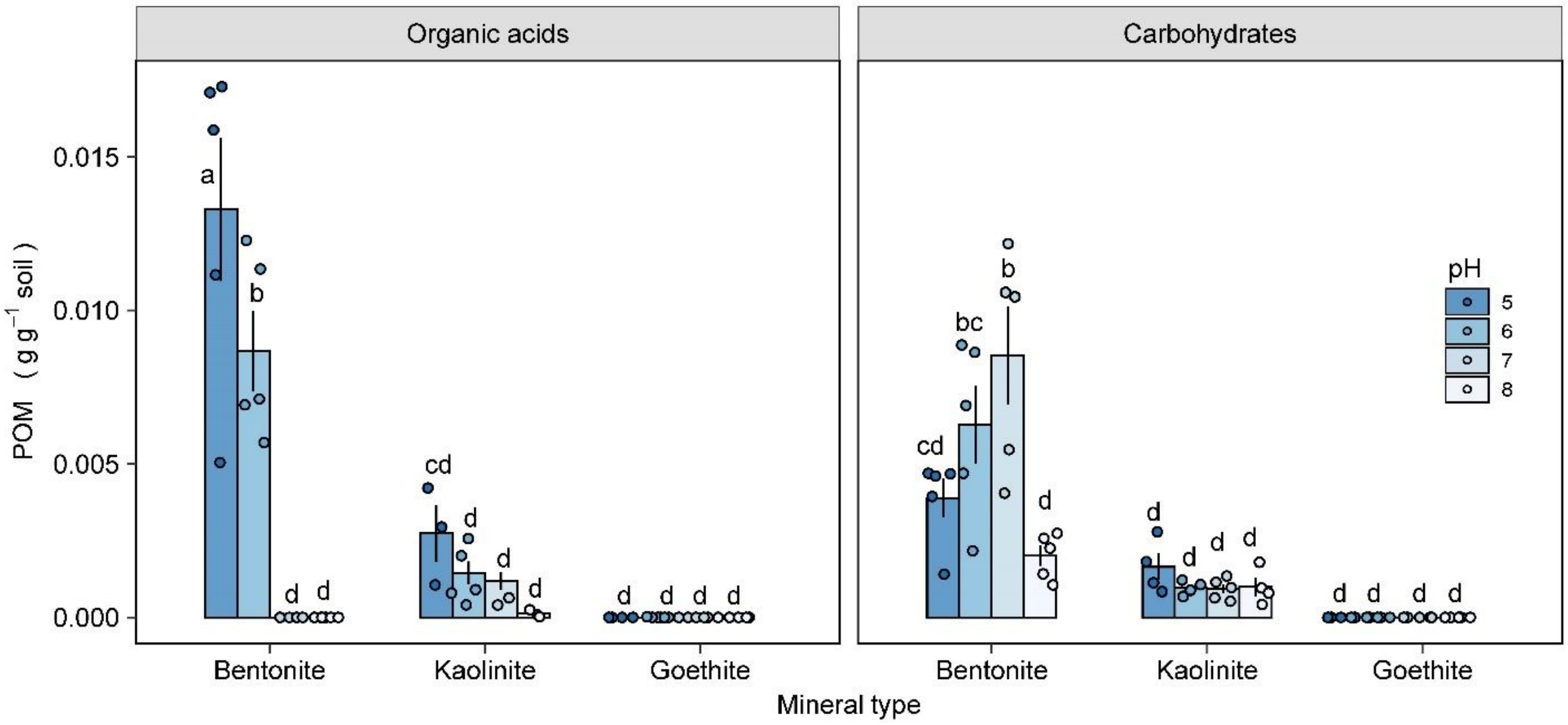

Fig. S17 Operationally recovered coarse microbial residues from the >53 μm fraction (sand removed by rinsing; residues captured on a 53 μm mesh) in different minerals across soil pH levels and root exudate treatments. Data are mean values ± se (n=5). Lower case letters indicate significant differences among different minerals, pH levels and root exudate types.

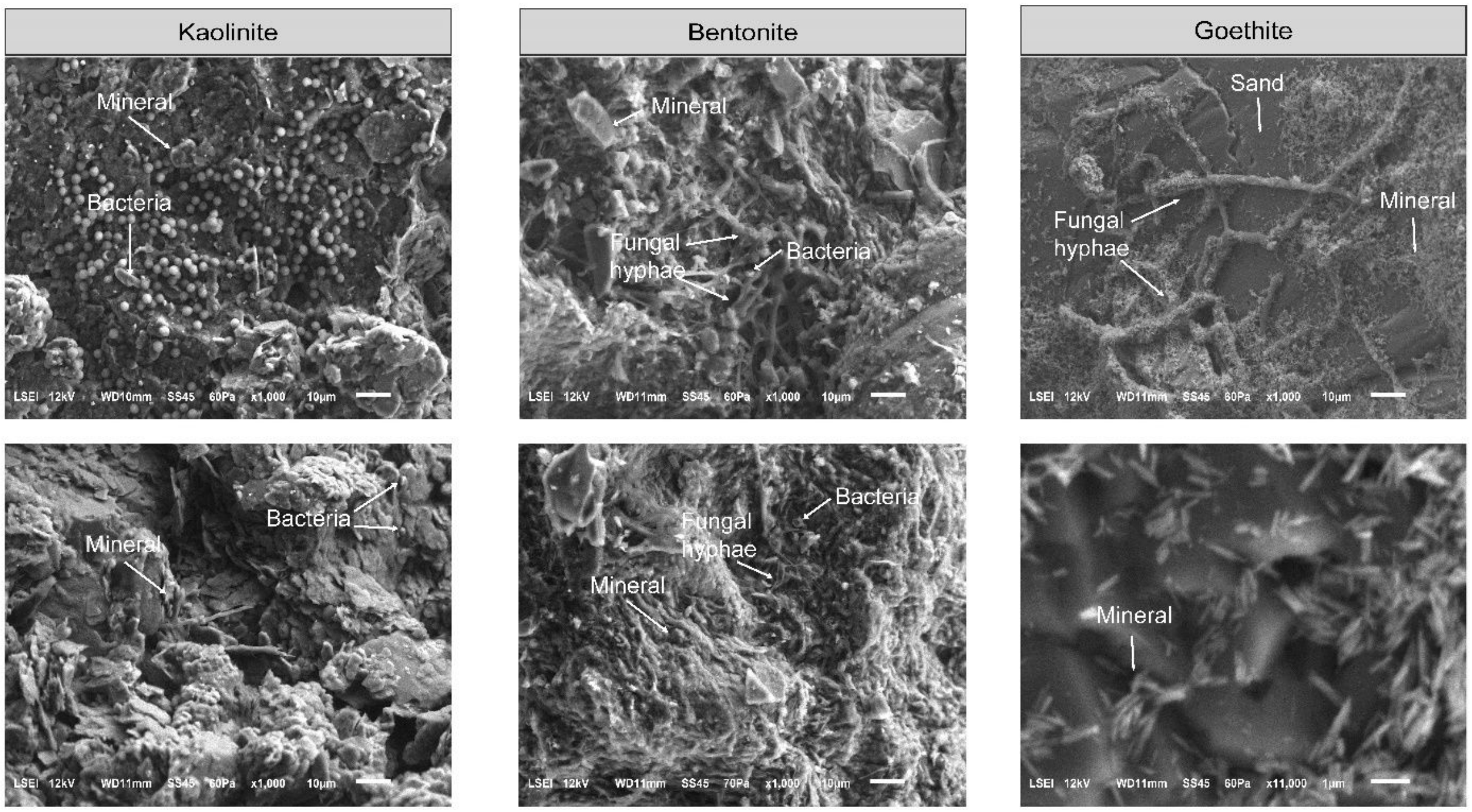

Fig. S18 Scanning electron microscopy (SEM) micrographs of the spatial connection of organo- mineral assemblages for different minerals, where microbial necromass are attached to the mineral surface. Those images were obtained from samples at pH=5 from carbohydrate treatment.
